# Drugs of abuse hijack a mesolimbic pathway that processes homeostatic need

**DOI:** 10.1126/science.adk6742

**Published:** 2024-04-19

**Authors:** Bowen Tan, Caleb J. Browne, Tobias Nöbauer, Alipasha Vaziri, Jeffrey M. Friedman, Eric J. Nestler

**Affiliations:** 1 Laboratory of Molecular Genetics, Howard Hughes Medical Institute, The Rockefeller University; New York, NY 10065, USA.; 2 Nash Family Department of Neuroscience, Friedman Brain Institute, Icahn School of Medicine at Mount Sinai; New York, NY 10029, USA.; 3 Brain Health Imaging Centre, Campbell Family Mental Health Research Institute, Centre for Addiction and Mental Health; Toronto, ON, M5T 1R8, Canada.; 4 Laboratory of Neurotechnology and Biophysics, The Rockefeller University; New York, NY 10065, USA.; 5 The Kavli Neural Systems Institute, The Rockefeller University; New York, NY 10065, USA.

## Abstract

Drugs of abuse are thought to promote addiction in part by “hijacking” brain reward systems, but the underlying mechanisms remain undefined. Using whole-brain FOS mapping and *in vivo* single-neuron calcium imaging, we found that drugs of abuse augment dopaminoceptive ensemble activity in the nucleus accumbens (NAc) and disorganize overlapping ensemble responses to natural rewards in a cell-type-specific manner. Combining FOS-Seq, CRISPR-perturbation, and snRNAseq, we identified *Rheb* as a molecular substrate that regulates cell-type-specific signal transduction in NAc while enabling drugs to suppress natural reward consumption. Mapping NAc-projecting regions activated by drugs of abuse revealed input-specific effects on natural reward consumption. These findings characterize the dynamic molecular and circuit basis of a common reward pathway, wherein drugs of abuse interfere with the fulfillment of innate needs.

## One-Sentence Summary

Convergent molecular, ensemble, circuit, and behavioral mechanisms mediate drug and natural reward interactions.

## Introduction

Drugs of abuse cause addiction by inducing persistent neuroplastic changes in brain reward circuits that have been evolutionarily established to direct behavior toward the satisfaction of need states such as hunger or thirst (1). Drug-induced changes in the function of these circuits narrows the scope of motivation toward their acquisition, which interferes with healthy goals (2, 3). Several theories of addiction development and maintenance depend on this idea (4, 5), and further imply that innate neural functions that normally process natural rewards are corrupted by drugs of abuse. However, neurobiological relationships between drug and natural rewards are typically inferred across studies and in separate experimental subjects, thus leaving the underlying physiological and molecular mechanisms linking these functions unclear. To address this gap, we compared the response of key reward circuits activated by food and water to responses to morphine or cocaine in the same animals.

## Results

### The nucleus accumbens is a central nexus of drug and natural reward

To establish a relationship between natural and drug reward processing, we determined how cocaine (a psychostimulant) and morphine (an opioid) affect behavioral responses to hunger and thirst (6). These studies were motivated by findings that individuals with substance use disorders exhibit marked deficits in appetite and nutrition (7). We first examined the effects of acute cocaine or morphine on feeding and drinking in fasted or dehydrated mice, using doses known to be rewarding (8) (Fig. S1a). Fasted mice that received either cocaine or morphine consumed less food during the first 30 min of refeeding compared to saline-treated mice, while morphine exerted a more prolonged suppressive effect on food intake that lasted 4 hours post-refeeding (Fig. S1b). In water deprived mice, acute cocaine or morphine exposure likewise decreased water intake during the 30-min rehydration period compared to saline, which recovered within 2–4 hours (Fig. S1b).

**Fig. 1.**
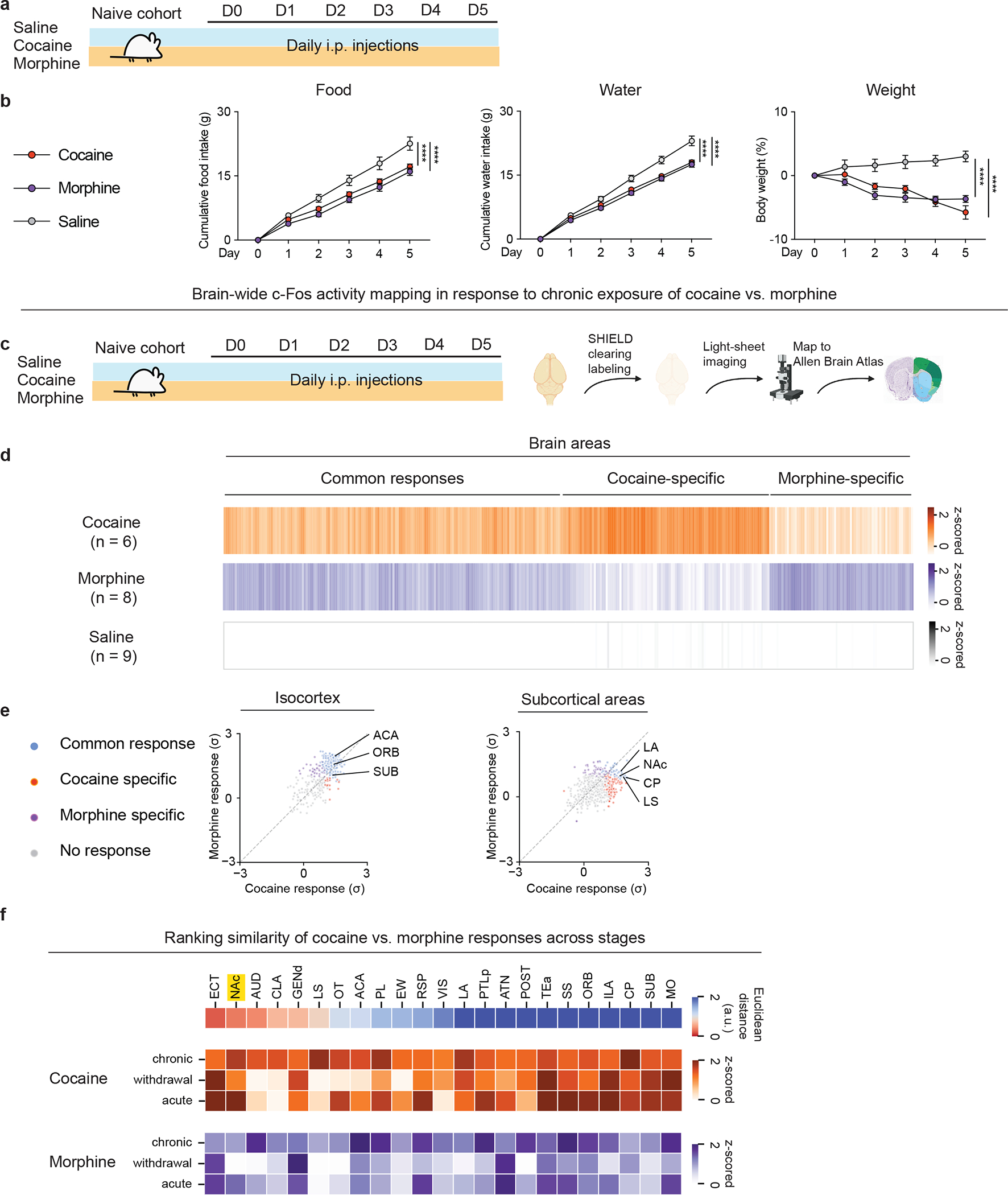
Whole-brain FOS mapping identifies shared and distinct brain regions activated by repeated exposure to cocaine and morphine. (a), Schematic of the experimental design for repeated exposure to drug rewards vs. saline. Comparisons of (b), Cumulative food intake (g), Cumulative water intake (g), Weight (%) over the 5-day treatment (n = 10, 10, 10 for saline, cocaine, morphine group, respectively, two-way ANOVA with Dunnett’s multiple comparisons). (c), Schematic of the experiment design for repeated exposure to drug rewards vs. saline followed by whole-brain clearing and mapping to Allen Brain Atlas. (d), Heatmap overview of brain areas showing significant FOS induction across three groups (One-way ANOVA for each brain area with cut-off p < 0.05 classified as statistically significant, followed by K-means clustering). (e), Scatter plot of FOS levels in cortical areas in response to cocaine vs. morphine (left). Scatter plot of FOS levels in subcortical areas in response to cocaine vs. morphine (right). Common response: areas showed significant changes (P < 0.05) of FOS+ counts in cocaine and morphine groups compared to the saline group; Cocaine or Morphine specific: areas only showed significant changes of FOS+ counts in either the cocaine or morphine group compared to the saline group. (f), Similarity of FOS responses across different phases of drug exposure (top). Heatmap representations of brain areas after acute or repeated exposure to cocaine or morphine or after spontaneous withdrawal (bottom). All error bars represent mean ± s.e.m. NS, not significant, *P < 0.05, **P < 0.01, ***P < 0.001, ****P < 0.0001.

We next measured the effects of repeated drug exposure on ad libitum food or water intake over the course of a five-day treatment regimen. Repeated daily exposure to either drug significantly reduced food and water intake, as well as body weight, compared to saline treatment (Fig. 1a, b). We tested whether withdrawal from repeated cocaine or morphine also affects behavioral responses to hunger and thirst. Mice were treated with cocaine, morphine or saline for 5 days, followed by saline administration for additional 3 days to produce spontaneous withdrawal (Fig. S1c). During this 3-day withdrawal, mice were fasted or water deprived overnight and then introduced into a new cage where they were provided free access to food for 20 min or water for 5 min. Mice with prior repeated cocaine or morphine exposure consumed less food or water compared to saline-treated mice (Fig. S1d). We also performed a sucrose preference test to assess anhedonic-like responses in withdrawal. While cocaine-treated mice showed normal sucrose preference, morphine-treated mice exhibited reduced sucrose preference (Fig. S1d).

We next identified putative brain regions that serve as sites of integration for neural responses to natural and drug rewards. We employed brain-wide FOS mapping after acute administration of either drug (Fig. 1c). Using a SHIELD-based whole-brain clearing approach (Fig. 1c), we observed three patterns of FOS activation: regions showing cocaine-specific responses, regions showing morphine-specific responses, and regions showing shared responses (Fig. 1d). Cortical regions activated by both drugs included three canonical addiction-related regions: anterior cingulate area (ACA), orbital area (ORB; orbitofrontal cortex), and subiculum (SUB). Among subcortical areas identified, the lateral amygdala nucleus (LA), nucleus accumbens (NAc), caudoputamen (CP), and lateral septum (LS) showed shared responses to both drugs (Fig. 1e). To identify brain regions showing an augmented response after multiple doses of morphine or cocaine, we performed additional section-based brain-wide FOS mapping experiments to compare activity across distinct phases of drug exposure: acute, repeated, and spontaneous withdrawal (Fig. S1e, f). Clustering analysis identified several brain areas showing increased FOS corresponding to each phase, with a subset of brain areas showing greater activation after acute vs. repeated drug treatment (Fig. S1e). These included the NAc, LA, claustrum (CLA), and others (Fig. 1f). This necessitated pair-wise comparisons of FOS from brain areas that show similar responses to both drugs across phases of drug exposure. Among hundreds of brain areas analyzed, the NAc showed increased FOS after both repeated and acute exposure to cocaine or morphine (Fig. 1f). Moreover, NAc was one of the top-ranked brain regions exhibiting analogous patterns of responses to cocaine vs. morphine across distinct phases.

The NAc houses two key subregions, core and shell, which exhibit unique cell-type composition, circuit projections, and functions in motivation and learning (3). We focused on the NAc core based on its well-established role in coordinating motivated behavior (9, 10). We stereotaxically delivered an inhibitory DREADD, AAV5-hsyn-hM4Di-mCherry, or a control AAV5-hsyn-mCherry bilaterally to the NAc, and three weeks later assessed food and water intake after combined treatment with clozapine N-oxide (CNO) plus cocaine, morphine, or saline (Fig. S2a). Chemogenetic silencing of NAc neurons prevented the cocaine- and morphine-induced reductions in food and water intake and body weight during repeated drug exposure (Fig. S2b). This chemogenetic silencing also blocked the suppressive effects of the drugs on refeeding and rehydration during spontaneous withdrawal (Fig. S2e, f). We next administered cocaine or morphine to these DREADD-expressing groups, and saline to the mCherry-expressing control group. 5-day repeated exposure to cocaine or morphine suppressed natural reward consumption and reduced body weight in the DREADD-expressing groups without CNO treatment (Fig. S2c, d). Silencing NAc neurons in the control group that received saline without drug exposure had no effect on food or water intake (Fig. S2g). Histological analysis confirmed that inhibitory DREADD expression was targeted mainly to the NAc core (Fig. S2h).

The NAc’s principal projection neurons NAc are medium spiny neurons (MSNs) which predominantly express either D1 or D2 dopamine receptors. These D1 and D2 populations exhibit distinct input-output architecture and are differentially engaged by drugs of abuse and natural rewards (11–13). We dissected the functional role of these two cell types in coordinating interactions between drug and natural rewards using an optogenetic approach in behavioral paradigms measuring key motivational outcomes. We recently demonstrated decreased food or water intake in a loss-of-function experimental setting, in which we optogenetically silenced these dopaminoceptive neurons in fasted or water-deprived mice (9). Our experimental objective here was to conduct a gain-of-function neural activation that emulated drug-induced effects on consummatory behaviors. We first stereotaxically delivered AAV5-hsyn-FLEX-ChR2 bilaterally in NAc of D1-Cre or D2-Cre transgenic mice, followed by implantations of optic fibers above the injection sites. We then optogenetically activated D1 or D2 MSNs in fasted mice with free access to food for 10 min, followed by 10 min laser off (Fig. S3a). Activation of D1 or of D2 MSNs potently decreased food intake in fasted mice during refeeding. Mice with prior activation of D2 MSNs showed compensatory overeating after laser stimulation, while this was not observed in mice with prior activation of D1 MSNs (Fig. S3a). Similarly, acute activation of D1 or D2 MSNs potently decreased water intake in dehydrated mice (Fig. S3b). In this case, we observed rebound water consumption after laser termination in both D1 and D2 mice (Fig. S3b).

We then tested the influence of D1 or D2 MSN activation on generalized locomotor activity. Activation of D1 MSNs substantially elevated locomotor activity in *ad libitum* fed mice, while activation of D2 MSNs substantially decreased locomotor activity (Fig. S3c) (13–16). We also tested whether activation of D1 or D2 MSNs conveyed valence signals independent of overall locomotion using real-time-place-preference (RTPP). Activation of D1 MSNs increased preference of animals for the stimulation side, indicative of D1 neurons conferring positive valence, while activation of D2 neurons caused an avoidance response to the stimulation side, indicative of conferring negative valence (Fig. S3d).

### Drugs and natural rewards activate an overlapping set of NAc neurons

**Fig. 2.**
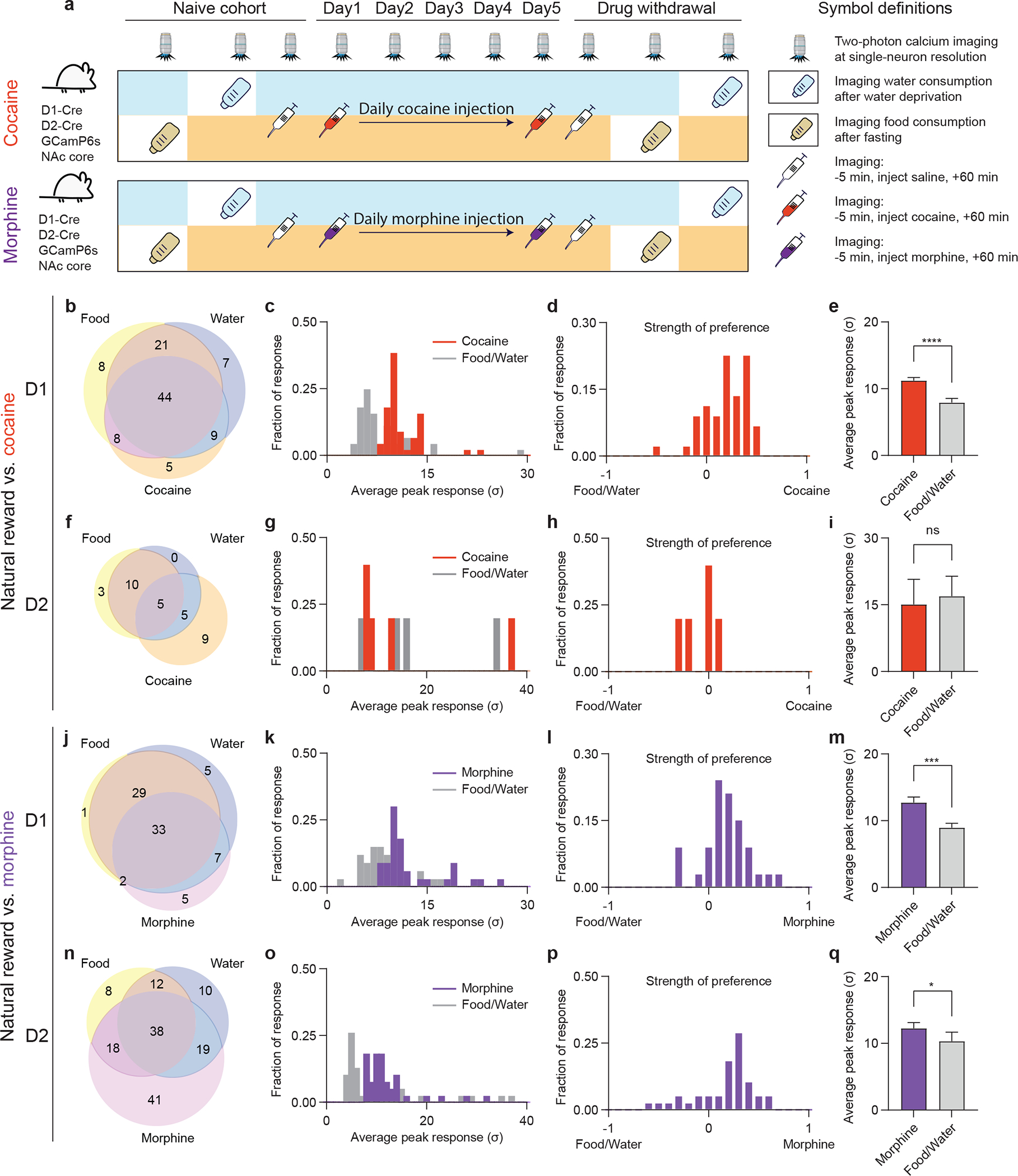
An overlapping set of dopaminoceptive neurons exhibit preferential activation in response to drugs of abuse over natural rewards. (a), Schematic of the experimental design for comparing neuronal responses to natural vs. drug rewards. (b), Venn diagram of D1 MSNs activated by food, water, or cocaine; n = 111 neurons pooled from 3 mice across all sessions recorded. (c), Distribution of average peak responses of D1 MSNs activated by food, water or cocaine. (d), Distribution of preferential activation strength of D1 MSNs between food/water vs. cocaine. (e), Comparison of peak responses of the neurons activated by food/water and cocaine (n = 44 neurons, two-tailed Wilcoxon test). (f), Venn diagram of activated D2 MSNs among food, water, cocaine; n = 46 neurons pooled from 3 mice across all sessions recorded. (g), Distribution of averaged peak responses of the D2 MSNs activated by food, water and cocaine. (h), Distribution of preferential activation strength of D2 MSNs between food/water vs. cocaine. (i), Comparison of the peak responses of the neurons activated by food/water and cocaine (n = 5 neurons, two-tailed Wilcoxon test). (j), Venn diagram of D1 MSNs activated by food, water, or morphine; n = 85 neurons pooled from 3 mice across all sessions recorded. (k), Distribution of average peak responses of the D1 MSNs activated by food, water and morphine. (l), Distribution of preferential activation strength of D1 MSNs between food/water vs. morphine. (m), Comparison of the peak responses of the above neurons activated by food/water and morphine (n = 33 neurons, two-tailed Wilcoxon test). (n), Venn diagram of D2 MSNs activated by food, water, or morphine. n = 170 neurons pooled from 3 mice across all sessions recorded. (o), Distribution of average peak responses of the D2 MSNs activated by food, water and morphine. (p), Distribution of preferential activation strength of D2 MSNs between food/water vs. morphine. (q), Comparison of the peak responses of the neurons activated by food/water and morphine (n = 38 neurons, two-tailed Wilcoxon test). All error bars represent mean ± s.e.m. NS, not significant, *P < 0.05, **P < 0.01, ***P < 0.001, ****P < 0.0001.

To determine whether drugs and natural rewards activate separable or overlapping populations of NAc neurons, we recorded the activity of individual neurons in response to food and water consumption vs. acute or repeated administration of cocaine or morphine. We directly tracked NAc D1 and D2 MSN activity at single-cell resolution using GRIN lens-based two-photon calcium imaging in headbar-fixed, treadmill-running mice (Fig. S4a, b) (9). Histological analysis confirmed that GCaMP6s expression was targeted mainly to NAc core (Fig. S4a). We first recorded individual neuronal responses during feeding and drinking after food or water deprivation, then before and after administration of drugs of abuse for 5 consecutive days, followed by imaging the same sets of neuronal responses to food and water during drug withdrawal (Fig. 2a). Responses of D1 and D2 MSNs to food and water consumption after a period of deprivation were similar to our previous report (9).

Cocaine administration elicited a pattern of D1 activation with a high proportion of overlap with those neurons activated by food or water (Fig. 2b). Only five out of a total of 111 detected D1 MSNs (4.5%) were activated by cocaine alone. To precisely compare the magnitudes of neural responses to food and water vs. cocaine, we quantified the Ca^2+^ transient peak amplitudes post food and water consumption vs. post cocaine administration. Among the D1 MSNs activated by all three types of rewards (i.e., food, water, and cocaine), the responding neurons were more potently activated by cocaine with significantly greater peak amplitudes (Fig. 2d, e). Cocaine also activated an ensemble of D2 MSNs, but a much smaller number compared to D1 neurons, with non-significant overlap with natural reward (Fig. 2f), and no difference in activation magnitudes (Fig. 2h, i).

There was also extensive overlap between morphine-activated D1 MSNs that responded to food or water (Fig. 2j) with only five out of a total of 85 D1 MSNs (6.2%) activated solely by morphine. For D2 MSNs, the overlap between morphine and natural rewards was partial, with morphine alone activating 41 neurons vs. 75 neurons that also responded to food or water (Fig. 2n). Morphine elicited a roughly equivalent cellular response in D1 and D2 MSNs, both of which exceeded responses to natural rewards (Fig. 2k–m).

Cocaine and morphine increase locomotor responses in mice, and striatal function controls both locomotor activity and reward processing (14, 16). Thus, to segregate reward-specific and locomotor-specific profiles of D1 and D2 responses, we compared the level of treadmill running to neural responses in cocaine- or morphine-responsive neurons to characterize motor- or nonmotor-associated (i.e., putative reward-processing) activity profiles (Fig. S4c, d). We made this assignment in a non-biased manner, but in retrospect noted clear anatomic differences in the focal plane between the motor- and nonmotor-associated ensembles (Fig. S4e). Using these classifications, we found that cocaine synchronized nonmotor-associated D1 MSNs (Fig. S4f, j) to a greater degree than motor-associated neurons (Fig. S4h, j), which only showed modest synchronization at 40 min (p=0.038). By contrast, cocaine did not synchronize the activity of D2 MSNs (Fig. S4f, j). On the other hand, morphine synchronized the nonmotor D2 MSNs (Fig. S4g, j), but not D1 MSNs (Fig. S4i, j). We also found a significant interaction effect between each drug reward and each cell type (Fig. S4j).

### Repeated drug exposure tunes cell-type-specific NAc dynamics

**Fig. 3.**
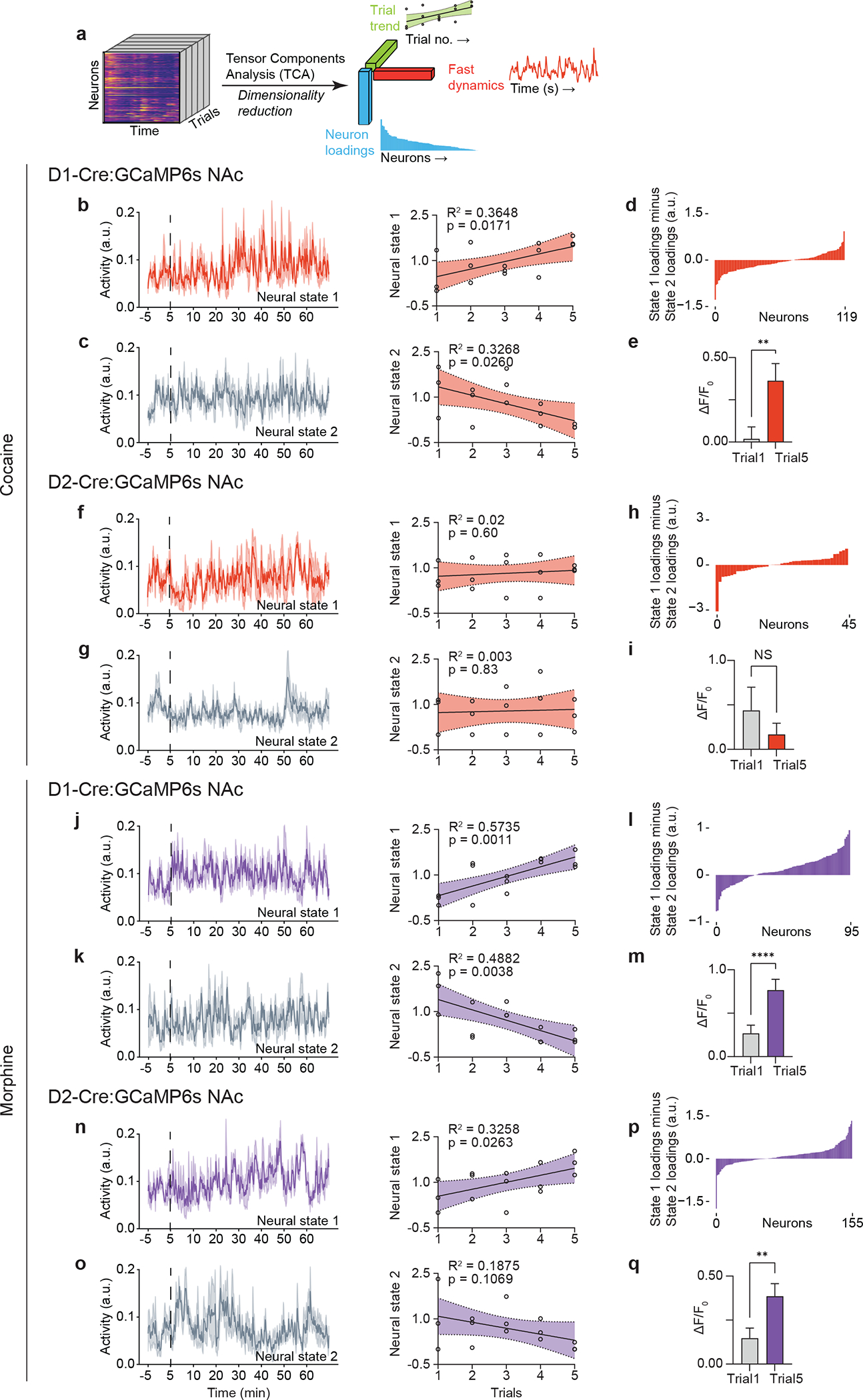
Repeated cocaine or morphine exposure augments the activity of subsets of dopaminoceptive neurons. (a), Schematic of tensor component analysis (TCA). (b), Cocaine-induced D1 neural state 1 and linear regression of the representation of neural state 1 across all sessions. (c), Cocaine-induced D1 neural state 2 and linear regression of the representation of neural state 2 across all sessions. (d), Loading factors of neurons contributing to state 1 relative to state 2 (n = 119 neurons merged from 3 mice across all 15 sessions). (e), Comparison of D1 MSNs positively contributing to cocaine-induced neural state 1 between session 1 and 5 (n = 46 neurons, two-tailed Wilcoxon test). (f), Cocaine-induced D2 neural state 1 and linear regression of the representation of neural state 1 across all sessions. (g), Cocaine-induced D2 neural state 2 and linear regression of the representation of neural state 2 across sessions. (h), Loading factors of neurons contributing to state 1 relative to state 2 (n = 45 neurons merged from 3 mice across all 15 sessions). (i), Comparison of D2 MSNs positively contributing to cocaine-induced neural state 1 between session 1 and 5 (n = 26 neurons, Wilcoxon). (j), Morphine-induced D1 neural state 1 and linear regression of the representation of neural state 1 across all sessions. (k), Morphine-induced D1 neural state 2 and linear regression of the representation of neural state 2 across all sessions. (l), Loading factors of neurons contributing to state 1 relative to state 2 (n = 95 neurons merged from 3 mice across all 15 sessions). (m), Comparison of D1 MSNs positively contributing to morphine-induced neural state 1 between session 1 and 5 (n = 68 neurons, Wilcoxon). (n), Morphine-induced D2 neural state 1 and linear regression of the representation of neural state 1 across all sessions. (o), Morphine-induced D2 neural state 2 and linear regression of the representation of neural state 2 across all sessions. (p), Loading factors of neurons contributing to state 1 relative to state 2 (n = 155 neurons merged from 3 mice across all 15 sessions). (q), Comparison of D2 MSNs positively contributing to morphine-induced neural state 1 between session 1 and 5 (n = 97 neurons, two-tailed Wilcoxon test). All error bars represent mean ± s.e.m. NS, not significant, *P < 0.05, **P < 0.01, ***P < 0.001, ****P < 0.0001.

Repeated drug exposure causes dynamic, cumulative plasticity within the NAc that has been suggested to contribute to behavioral abnormalities underlying addiction (1, 17–20). To examine whether repeated exposure to cocaine or morphine causes neuroplastic changes within ensembles of D1 and D2 neurons, we utilized tensor component analysis (TCA, Fig. 3a), which reduces and organizes multi-trial neuronal dynamics into lower-dimensional factors, effectively subgrouping the responding neurons based on their within-trial dynamics and across-trial evolution (21). A subset of D1 MSNs showed amplified responses to cocaine over five days (Fig. 3b), while a separate group of D1 MSNs that did not respond to acute cocaine displayed decreasing activity over the course of drug treatment (Fig. 3c). Repeated exposure to cocaine did not show an amplifying effect on D2 MSN activity (Fig. 3f, g). In contrast, repeated morphine exposure amplified subsets of both D1 and D2 neural responses over time (Fig. 3j, n), while, similar to cocaine, the inactive D1 neural cluster showed a decreasing trend over trials (Fig. 3k). The distinct augmentation of neural responses induced by repeated exposure to cocaine vs. morphine identifies drug-specific – potentially pathological –changes in D1 vs. D2 MSNs within NAc. This shift in ensemble dynamics was not observed throughout serial testing with natural rewards (9).

### Withdrawal from drug exposure disorganizes neural responses to natural rewards

**Fig. 4.**
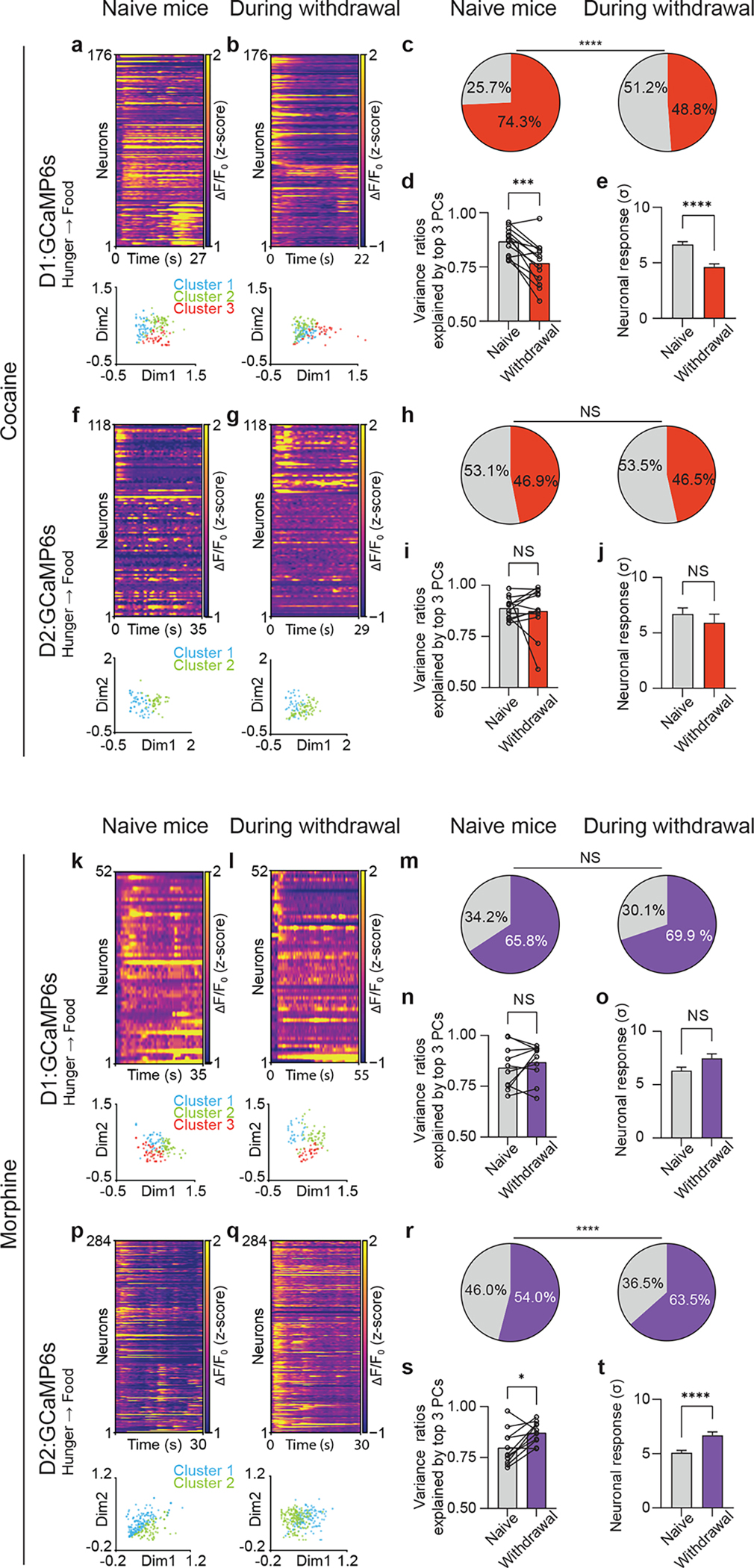
Disorganized cell-type-specific responses to natural rewards following cocaine or morphine withdrawal. (a, b) Representative heatmaps of D1 neuronal responses (top panels) to food prior to cocaine exposure or following cocaine withdrawal (n = 176 matched neurons), and non-negative matrix factorization (NMF) representation of neuronal states labeled by k-means clustering (bottom panels) (c), Percentage of D1 MSNs activated by food or water (n = 914 matched neurons, 13 matched sessions, 3 mice). (d), Variances explained by top 3 principal components (PCs) during food and water consumption (n = 13 matched sessions, 3 mice). (e), D1 responses to food and water (n = 914 matched neurons). (f, g) Representative heatmaps of D2 responses to food prior to cocaine exposure or following cocaine withdrawal (n = 118 matched neurons), and NMF representation of neuronal states. (h), Percentage of D2 MSNs activated by food or water (n = 488 neurons, 12 sessions, 3 mice). (i), Variances explained by top 3 PCs during food and water consumption (n = 12 sessions, 3 mice). (j), D2 responses to food and water pre/post cocaine (n = 488 matched neurons). (k, l), Representative heatmaps of D1 responses to food prior to morphine exposure or following morphine withdrawal (n = matched 52 neurons), and NMF representation of neuronal states. (m), Percentage of D1 MSNs activated by food or water pre/post morphine (n = 587 matched neurons, 10 sessions, 3 mice). (n), Variances explained by top 3 PCs during food and water consumption (n = 10 sessions, 3 mice). (o), D1 responses (n = 587 matched neurons). (p, n), Representative heatmaps of D2 responses to food prior to morphine exposure or following morphine withdrawal (n = 284 matched neurons) (n), and NMF representation of neuronal states. Percentage of D2 MSNs activated by food or water (n = 1174 neurons, 11 sessions, 3 mice). (s), Variances explained by top 3 PCs during food and water consumption (n = 11 sessions from 3 mice). (t), D2 responses to food and water (n = 1174 neurons). The Fisher’s exact test was used to compare the percentages of neurons. The two-tailed paired t-test was used to compare variance ratios. The two-tailed Wilcoxon test was used to compare the neuronal responses. Error bars represent mean ± s.e.m. NS, not significant, *P < 0.05, **P < 0.01, ***P < 0.001, ****P < 0.0001.

A hallmark of addiction is the propensity for relapse after periods of abstinence, suggesting that prior consumption of drugs of abuse causes protracted disruptions in reward processing. We therefore examined whether withdrawal from repeated drug exposure interferes with homeostatic responses to food or water. Two days after the last drug injection (i.e., on day 7), mice were fasted (or dehydrated) and allowed to consume food (or water), while the same D1 or D2 MSNs were imaged. During refeeding three distinct clusters are activated during the consummatory phase correlating with meal initiation, continued feeding, and meal cessation (9). Here, however, we found that during cocaine withdrawal these three D1 neuronal clusters were entirely disordered with significantly reduced variances. This change was captured by a reduced percentage of activated D1 MSNs and a diminution of correlated neuronal activity, with the reduced variance ratio explained by the top three principal components (PCs) representing the structure of neural dynamics (Fig. 4a–e). However, cocaine withdrawal did not alter D2 neuronal responses to natural rewards (Fig. 4f–j). In contrast, during morphine withdrawal, D2 MSNs showed markedly increased activation with increased variance ratio explained by the top three PCs (Fig. 4p–t), while morphine withdrawal did not alter the responses of D1 MSNs (Fig. 4k–o).

### *Rheb* mediates drug-induced interference of natural reward processing

**Fig. 5.**
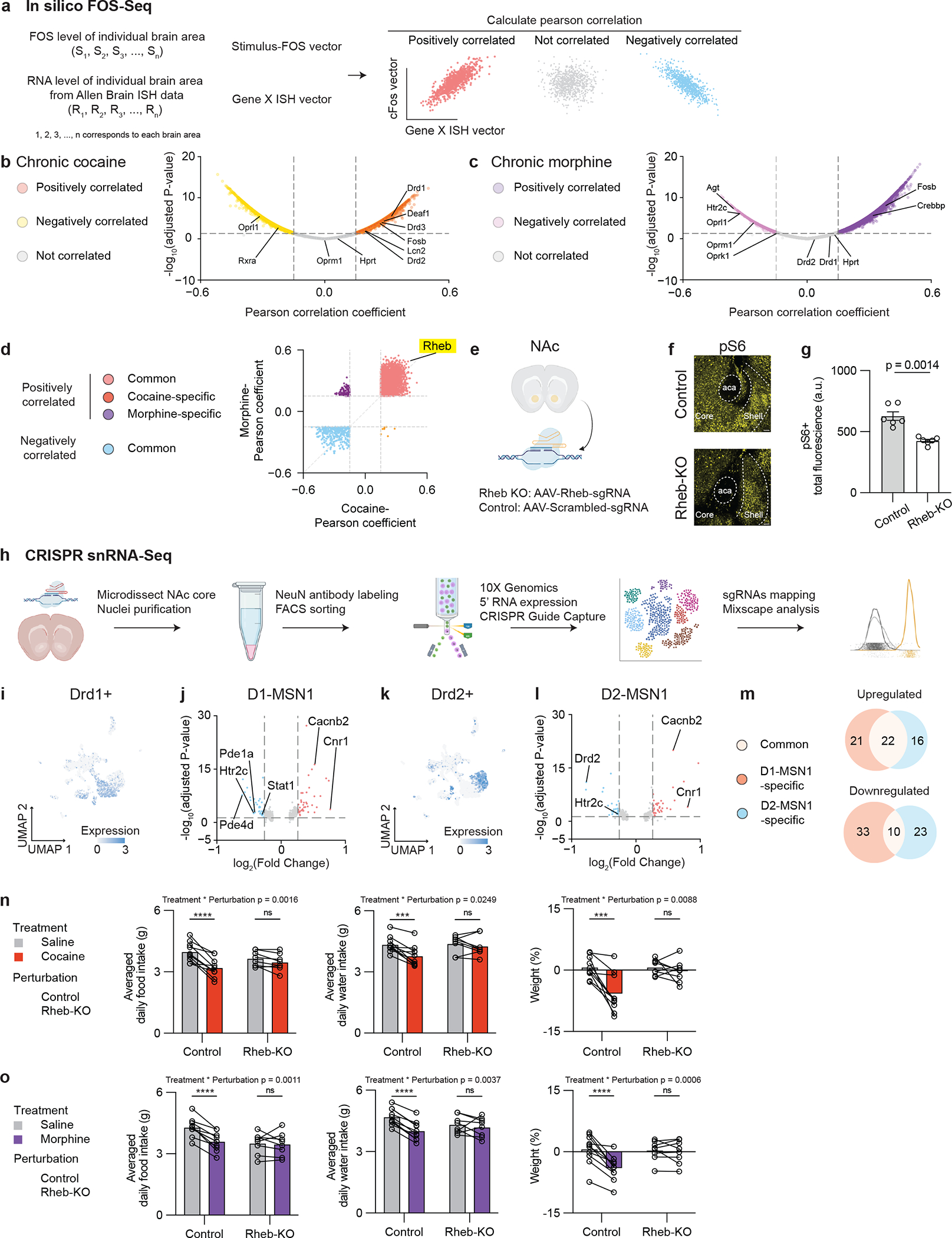
*Rheb* regulates cell-type-specific signal transduction and is necessary for the ability of repeated cocaine and morphine exposure to suppress natural reward consumption. (a), Schematic of the *in silico* FOS-Seq approach to identify genes associated with brain-wide FOS patterns. Genes with Pearson Correlation Coefficient > 0.15 or < −0.15 and p < 0.05 are classified as positively correlated or negatively correlated genes. Adjusted P-values are FDR-corrected at 5% threshold. (b), Volcano plot of genes associated with repeated exposure to cocaine. (c), Volcano plot of genes associated with repeated exposure to morphine. (d), Scatter plot of Pearson coefficient from genes associated with repeated exposure to cocaine vs. Pearson coefficient from genes associated with repeated exposure to morphine. (e), Schematic of *in vivo* NAc region-specific knockout of *Rheb* gene by co-expressing Cre and Rheb-sgRNAs or their control scrambled-sgRNAs in NAc core in LSL-Cas9 transgenic mice. (f), Immunohistochemistry validation of pS6 levels in the NAc from the *Rheb* knockout (*Rheb*-KO) group vs. the Control group at baseline (scale bar: 100 μm). (g), Quantification of total pS6 fluorescent intensity in the NAc (n = 6 sections per group, with 2 sections per animal, 3 animals per group. The 2 sections were each chosen from anterior and posterior NAc, with at least 200 μm apart). (h), Schematic of snRNA-seq after CRISPR perturbations (n = 7001 cells mapped with either sgRNA). (i), Distribution of *Drd1*+ cells in the UMAP. (j), Differentially expressed genes in *Rheb*-KO cells vs. control cells in the D1-MSNs1 cluster (n = 598, 390 cells respectively). (k), Distribution of *Drd2*+ cells in the UMAP. (l), Differentially expressed genes in *Rheb*-KO cells vs. control cells in the D2-MSNs1 cluster (n = 449, 375 cells respectively). (m), Venn diagram of genes significantly regulated by *Rheb*-KO between D1-MSNs1 and D2-MSNs1 clusters. (n, o), Comparisons of 5-day averaged daily food intake (g), and daily water intake (g), as well as weight (%) after 5-day drug exposure in the *Rheb*-KO group treated with saline for 5 days followed by another 5-day cocaine or morphine treatment (n = 10, 8 for the Control and *Rheb*-KO groups, respectively. Two-way ANOVA with Šídák’s multiple comparisons). All error bars represent mean ± s.e.m. NS, not significant, *P < 0.05, **P < 0.01, ***P < 0.001, ****P < 0.0001.

These calcium imaging findings establish a model of neural dynamics wherein drugs of abuse modulate the activity of individual NAc neurons that normally process physiological needs. To explore the potential mechanisms by which drugs alter neural dynamics, we developed an *in silico* approach termed “FOS-Seq” that leverages the aforementioned brain-wide activity mapping data to search for potential molecular substrates essential for the effects of drug rewards on food and water consumption (22). We computationally compared the anatomic distribution of FOS to that of individual genes in the Allen Brain Atlas and computed Pearson Correlation Coefficients (PCC) between brain-wide FOS activity vectors and individual *in situ* expression vectors (ISH vectors) for each gene (Fig. 5a and Fig. S5a) (23). This approach reliably captured canonical marker genes temporally associated with the addicted state (Fig. 5b, c and Fig. S5b, c). Specifically, after repeated exposure to cocaine, we identified a positive correlation between FOS and *Drd1*, *Drd2*, *Drd3*, *Deaf1*, *Fosb,* and *Lcn2* expression (Fig. 5b). Additionally, the *Oprl1* and *Rxra* genes were negatively correlated with FOS, while the *Oprm1* gene was not correlated with FOS (Fig. 5b). In contrast, repeated exposure to morphine elicited positive correlations between FOS and *Fosb* and *Crebbp* (Fig. 5c). We also found a negative correlation between FOS and *Agt*, *Htr2c*, *Oprl1*, *Oprm1,* and *Oprk1* expression (Fig. 5c). By contrast, the reference “housekeeping” gene serving as a negative control, *Hprt*, was not significantly correlated with either group (Fig. 5b, c). To validate this *in silico* approach, we conducted the same analysis for FOS activity vectors from acute exposure and spontaneous withdrawal of cocaine or morphine. Again, this approach consistently captured canonical marker genes known to show changes in gene expression in response to each of the two drugs of abuse (Fig. S5d, e). To directly identify genes from FOS-Seq that are shared between repeated cocaine and morphine exposure, we generated a scatter plot of PCCs for each gene by analyzing the intersection among those that were significantly correlated with FOS (Fig. 5d). We found *Rheb* as a top-ranked gene positively correlated with FOS, and uniquely shared by repeated exposure to cocaine or morphine (Fig. 5d). *Rheb* encodes a GTP-binding protein that phosphorylates mTOR and activates downstream pathways (24). Several other genes in the mTOR pathway are likewise significantly correlated with FOS activity (Fig. S5c, d) (31–36).

Because we found that that inhibiting NAc neurons blunts the ability of drugs of abuse to suppress natural reward consumption (Fig. S2), we tested whether *Rheb* may have a role in coordinating this effect. We generated a region-specific knockout of *Rheb* or its control by stereotaxically delivering AAV5-hsyn-Cre and AAV5-Rheb-sgRNAs-hsyn-mCherry or AAV5-control-sgRNAs-hsyn-mCherry bilaterally in NAc core of LSL-Cas9 transgenic mice (Fig. 5e). We first immunohistochemically probed levels of phospho-S6 (pS6; a ribosomal protein), a well-established marker for RHEB-mTOR activity (25), in the NAc of the *Rheb* knockout and control mice (Fig. 5f). The *Rheb* knockout (Rheb-KO) group showed decreased pS6 fluorescence in the NAc core compared to the control group (Fig. 5f, g, Fig. S7e), suggesting a high knockout efficiency of *Rheb* with this CRISPR approach. We conducted single-nucleus RNA-sequencing (snRNAseq) from neurons in NAc core and mapped those transduced with sgRNAs post hoc to examine cell-type-specific effects of *Rheb* knockout (Fig. 5h). snRNAseq of NAc core revealed 18 different clusters that were marked by single or double molecular markers (Fig. S6a, b). D1 (*Drd1*+) and D2 (*Drd2*+) cells were spatially segregated in the UMAP (Fig. 5i, k). Among these cells, D1 MSNs were further comprised of three subtypes: cluster1 *Drd1/Pdyn* (D1-MSN1), cluster5 *Drd1/Tshz1* (D1-MSN2), and cluster11 *Drd1/Drd3* (D1-MSN3); D2 MSNs were also comprised of three subtypes: cluster2 *Drd2/P2ry1* (D2-MSN1), cluster7 *Drd2/Reln* (D2-MSN2), and cluster14 *Drd2/Htr7* (D2-MSN3).

Using our snRNAseq results, we evaluated how *Rheb* knockout affected D1 and D2 MSNs. Prior reports show that *in vivo* CRISPR perturbation induces both frame-shift and in-frame mutations, thus resulting in subsets of cells that escape perturbations (26, 27). To parse these ‘escaped’ cells, we applied Mixscape analysis (26), a validated algorithm that classifies *Rheb*-sgRNAs+ cells into perturbed and escaped cells compared to Control-sgRNAs+ cells (Fig. S6c). Perturbed cells were specific within D1-MSN1 and D2-MSN1 clusters, two main subtypes of D1 and D2 MSNs (Fig. S6d). We next performed differential gene expression analysis on perturbed vs. control cells from D1-MSN1 and D2-MSN1 clusters (Fig. 5j, l). *Rheb* knockout decreased expression of *Htr2c*, which encodes the 5HT2C serotonin receptor, and increased expression of *Cacnb2*, which encodes a voltage-gated calcium channel subunit, as well as *Cnr1*, which encodes the CB1 cannabinoid receptor, in both D1-MSN1 and D2-MSN1 clusters (Fig. 5j, l). In contrast to this common set of genes, *Rheb* knockout in D1-MSN1 specifically decreased *Pde1a* and *Pde4d*, which encode phosphodiesterase enzymes associated with cAMP signal transduction. In D2-MSN1, *Rheb* knockout decreased expression of *Drd2* (Fig. 5j, l). Overall, *Rheb* knockout in D1-MSN1 and D2-MSN1 led to transcriptional regulation of both common and distinct subsets of genes (Fig. 5m). Gene ontology analysis revealed that distinct pathways within each cell type were affected by *Rheb* knockout (Fig. S6e).

Considering that *Rheb* knockout produces transcriptomic consequences that are associated with drug signaling within the NAc core, we examined whether *Rheb* has a causal role in mediating the behavioral effects of drugs of abuse on natural reward consumption. Mice received viral transduction of *Rheb*-sgRNAs or control-sgRNAs in NAc core, and were subsequently administered saline for 5 days, followed by an additional 5 days of repeated exposure to cocaine or morphine. In the knockout group, cocaine and morphine no longer decreased food or water intake or body weight compared to prior saline treatment in the same animals (Fig. 5n, o, Fig. S7b, d). In contrast, the control-sgRNA group consistently showed reduced consumption of natural rewards by both drugs (Fig. 5n, o, Fig. S7a, c). We also observed a significant interaction effect between treatment (saline vs. cocaine or morphine) and perturbation (Control vs. Rheb-KO) (Fig. 5n, o).

### Identification of NAc inputs for integration of natural and drug reward

The NAc receives inputs from numerous regions that carry information relevant to reward processing across drug classes (11, 28–30). In contrast, there are few direct inputs from brain regions that sense physiological needs such as arcuate nucleus neurons that regulate feeding or subfornical organ neurons that regulate fluid intake (31–33). We set out to identify how signals reflecting physiological needs relay to the NAc. We delivered rAAV2-retro-CAG-GFP virus unilaterally to the NAc to label monosynaptic inputs. Mice then received repeated injections of cocaine, morphine, or saline (Fig. S8 and Fig. S9), followed by SHIELD-based whole-brain mapping of FOS and GFP expression in the same brain. This enabled us to overlay sites of FOS activation with sites that project monosynaptically to NAc and express GFP from the retrograde NAc tracer. We then overlaid GFP density and FOS activity in the same brain in response to each stimulus (Fig. S8 and Fig. S9). Lateral septum (LS), subiculum (SUB), anterior dorsal thalamus (ATN), prefrontal cortex (ORB, ILA, PL, and ACA), ectorhinal area (ECT), and amygdala (LA, CLA) showed FOS after both cocaine and morphine treatment while also showing strong retrograde GFP signals from NAc (Fig. S10a–c). To examine the causal roles of these nodes, we conducted gain-of-function chemogenetic activation experiments. We delivered rgAAV-hSyn-DIO-hM3D(Gq) into NAc core, and delivered AAV5-hSyn-Cre to different mouse groups (except the control group) in these areas: LS, ORB, ventral SUB (vSUB), ATN/aPVT, ACA, LA/BLA, medial PFC (mPFC: ILA, PL, Cg1), or CLA/ECT (Fig. S10d, e). This combined retrograde-DREADD and Cre-labeling strategy enables specific expression of an activating-DREADD in NAc-projecting neurons in each of these areas. CNO was administered to each group of mice, and food or water consumption was measured. Activation of LS or ORB neurons among these 8 NAc-projecting nodes decreased food consumption (Fig. S10d). Moreover, only the activation of ORB neurons decreased water consumption (Fig. S10d).

## Discussion

### Divergent cellular dynamics link drug and natural reward processing

Our ensemble imaging data revealed that cocaine and morphine more robustly activate neurons within NAc that also respond to natural rewards in a cell-type-specific manner. Repeated exposure to these drugs also caused progressive tuning of response patterns, which contrasts with the fixed neural responses to repeated consumption of palatable food or water (9). These results imply that drug exposure dysregulates a highly coordinated system that normally matches physiological need to appetitive behavior. We found further that cocaine- and morphine-induced changes in NAc cellular dynamics differentiated their preferential actions across D1 and D2 MSNs. Consistent with evidence from previous studies (34), we observed that cocaine almost exclusively activates D1 MSNs and amplifies their activity with repeated exposure, while morphine activates and amplifies both D1 and D2 MSN function. This cell-type-specific evolution of NAc dynamics could reflect divergent neural substrates that coordinate separable responses across drug classes and that are computed in the same brain region to enhance the reward value of each drug while interfering with value processing for natural rewards. In addition to physiological symptoms, withdrawal from drug intake produces dysphoria and negative affect, which potentially reflective a rebound from positive to negative valence. D2 MSN activation can promote aversive effects, while D1 activation promotes positive reinforcement (12, 13, 35). Therefore, the diminished D1 neuronal responses to natural rewards observed during withdrawal from cocaine may reflect an interference with appetitive behavior, while augmented D2 neuronal responses during morphine withdrawal may reflect a shift toward negative affect-based seeking. Thus, our results point to partly separable neuronal coding principles across drug classes with direct relevance to addiction and withdrawal.

### *Rheb* links drug exposure with natural reward processing

Repeated drug exposure drives molecular changes in NAc which are thought to underlie long-term plasticity. Numerous studies have implicated cell-type-specific molecular processes within NAc that mediate the addictive actions of drugs of abuse (36). One example is ΔFOSB, whose D1 MSN-specific induction by chronic cocaine and combined D1 and D2 MSN induction by chronic opioids mirrors the patterns of NAc neuronal activation observed here (37). Interestingly, ΔFOSB is also induced by high levels of consumption of natural rewards and this induction in turn increases such consumption (38). However, this mechanism is self-limited for natural rewards, which do not show the amplification of signals over time as do drugs of abuse. This raises the question of whether a specific molecular substrate(s) within NAc drives the pathological effects of drug exposure while simultaneously interfering with innate responses to natural rewards. Our study identifies such a substrate—the mTOR activator, RHEB—as a crucial intermediate specifically linking the ability of drug exposure to attenuate food and water consumption. Sustained mTOR activation in NAc by repeated drug exposure might thus facilitate the development of neural plasticity during the pathogenesis of addiction. Previous studies have shown that reducing mTOR activity in NAc with rapamycin, a potent mTOR inhibitor, inhibits cue-induced reinstatement of cocaine-seeking without affecting cue-induced sucrose seeking behavior, indicating a specific role for mTOR activity in drug action (39–41). However, these effects appear to be region-specific as reducing mTOR activity in ventral tegmental area mediates morphine tolerance (42). Single-cell sequencing and spatial transcriptomics have revealed considerable cellular heterogeneity within NAc (43–47), but it remains unknown how mTOR signaling controls divergent actions of cocaine and morphine. One possibility, suggested by our imaging and snRNAseq results after *Rheb* knockout, is that cocaine vs. opioid rewards preferentially sensitize firing rates of distinct cell types, and that the intracellular mTOR pathway is then essential for each cell-type-specific drug action. This raises an important future line of research to understand how a generic signal transduction pathway distinguishes between rewarding stimuli and adapts neural sensitivity accordingly (48–50). Further, the development of new tools that enable combined *in vivo* single-neuron calcium imaging with CRISPR perturbations will be crucial to resolving how RHEB-mTOR signaling processes both appetitive and aversive aspects of motivation at the level of neural dynamics (51). Answering these fundamental questions could potentially lead to development of cell-type-specific mTOR modulators that would enhance addiction treatment options.

### Drugs and natural rewards engage similar NAc inputs

The NAc is an integrative node in the limbic circuitry that is essential for reward processing and generating motivated behavior. These processes require integration of complex interoceptive and exteroceptive signals, information relevant to learned associations among sensory stimuli, reward acquisition, and anticipatory and coordinated motoric output centers. It is therefore likely that specific input/output networks connected with NAc influence the specific information being processed within the NAc. While our results suggest a role for the NAc in sensing hunger and thirst, the NAc receives very few direct ascending inputs from canonical energy and fluid homeostasis nodes such as the hypothalamic arcuate nucleus or subfornical organ, respectively (32, 52). We used a whole-brain approach, which provided a comprehensive map of ascending nodes activated by both cocaine and morphine that project to NAc, including prefrontal cortex, LA, anterior thalamic nuclei, and LS. Prior literature implicated several of these regions in both natural and drug reward processing, from homeostatic intake to roles in coordinating conditioned food- or drug-seeking behavior (52–56). By activating several of these pathways, we identified a particularly important role for ORB inputs to NAc in coordinating consumption of natural rewards. While we focus on ORB, it is likely that other NAc inputs have important roles in other behavioral domains (57–60). ORB is crucial for decision making and updating reward value based on internal states (61, 62). Thus, our findings suggest that value information communicated to NAc through ORB has a broad influence on consummatory behaviors that fulfill homeostatic need. These results support and extend numerous previous reports that ORB is crucial for evaluation and preference of drug vs. nondrug rewards, potentially by computing reward identity (63–65). Because drugs of abuse can cause persistent changes to ORB function (66), it is plausible that this ORB-NAc circuit is crucial for drugs of abuse to modulate valuation and decision making in goal-directed behaviors (66, 67).

Together, these results point to the NAc as a gateway for integrating and amplifying the value of drug rewards, and suggest that this action serves as a prime regulator of behavior to ultimately promote drug-seeking and drug-taking. However, it is important to note that our studies utilize non-volitional drug exposure to link neural ensembles and molecular substrates to volitional food and water intake. Studies employing instrumental tasks that directly compare motivation for drugs of abuse with natural reward in rodents suggest that drugs may have stronger motivational properties than natural rewards (68–70), but may not be strongly preferred over natural reward when given the choice (69, 71–76). Unravelling these relationships at the neurophysiological and molecular levels will be crucial for insights into the mechanisms governing shifts in goal-directed behavior during the development of addiction (77, 78). Employing drug self-administration within the framework outlined in our study will be important to determine whether volitional drug intake induces similar changes at circuit, ensemble, and molecular levels (79). Further, determining the role of neural mechanisms outlined here in mediating interactions between drugs of abuse and other non-homeostatic goals, particularly socially motivated stimuli and hedonic rewards (4, 80, 81), will be necessary to understand the full sequelae of addiction.

## Materials and Methods

### Mouse strains

Male adult mice (8–24 weeks old) were used for experiments. We obtained Drd1-Cre mice (Drd1-Cre120Mxu/Mmjax, stock no. 37156), wild-type mice (C57BL/6J, stock no. 000664), and LSL-Cas9 mice (stock no. 026175) from the Jackson Laboratory. Drd2-Cre mice were obtained from Dr. E. Azevedo at The Rockefeller University as previously described (82). All mice were maintained in temperature- and humidity-controlled facilities on a 12-h light-dark cycle (light on at 7:00 am) and had ad libitum access to food and water except when noted otherwise. All feeding experiments used standard rodent chow pellets. For fasting or dehydration experiments, mice were overnight fasted or dehydrated (16–24 hours of food or water deprivation). All experimental protocols were approved by the IACUC at the Rockefeller University and at Mount Sinai, according to the NIH Guide for the Care and Use of Laboratory Animals.

### Viral vectors

The following AAV viruses were purchased from the vector core at the University of North Carolina: AAV5-EF1a-DIO-ChR2-YFP, AAV5-EF1a-DIO-YFP, AAV5-EF1a-DIO-mCherry. The following AAV viruses were purchased from Addgene: AAV5-hsyn-hM4Di-mCherry or AAV5-hsyn-DIO-hM4D(Gi)-mCherry, AAV5-hsyn-mCherry, rAAV2-retro-CAG-GFP, AAV5-hsyn-Cre, AAV5-hsyn-eGFP-Cre, AAV1-hSyn-FLEX-GCaMP6s, AAVrg-hSyn-hM3D(Gq)-mCherry. The plasmid expressing guide RNAs targeting *Rheb*: pAAV-U6-sgRNA1[Rheb]-U6-sgRNA2[Rheb]-hsyn-mCherry, and non-targeted control: pAAV-U6-sgRNA1[Scrambled]-U6-Rheb-sgRNA2[Scrambled]-hsyn-mCherry were designed and engineered at VectorBuilder. The plasmids were packaged into AAV5 at Janelia Viral Tools. sgRNA1[Rheb] sequence: ACCAAGTTGATCACGGTAAA, sgRNA2[Rheb] sequence: GTTCTCTATGGTTGGATCGT. sgRNA1[Scrambled] sequence: GTGTAGTTCGACCATTCGTG, sgRNA2[Scrambled] sequence: GTTCAGGATCACGTTACCGC.

### Stereotaxic surgery

Mice were induced with 3% inhaled isoflurane anesthetic in oxygen, placed in a stereotaxic apparatus (Kopf Instruments) and maintained at 1.5% isoflurane during surgery. The coordinates of injection sites were determined by the website tool: http://labs.gaidi.ca/mouse-brain-atlas. All AAV viruses were diluted in 1X PBS to a titer range of 3 to 8 × 10^12^ GC/ml before use. Viruses were then bilaterally injected in the nucleus accumbens core (300–500 nl per side, 100 nl/min) using the following coordinates relative to the bregma: AP: +1.32; ML: ±1.1; DV: −4.25–4.5. For optogenetic experiments, optic fibers with 200-μm diameter core (Thorlabs CFML12U-20) were placed 0.3–0.5 mm above the virus injection site. For in vivo two-photon imaging surgeries, in order to obtain sufficient expression of GCaMP6s, 600 nl of virus were delivered (300 nl per coordinate) at AP: +1.22; ML: +1.2; DV: −4.25 and AP: +1.42; ML: +1.2; DV: −4.25 or at AP: +1.32; ML: +1; DV: −4.25 and AP: +1.32; ML: +1.3; DV: −4.25. A gradient-index (GRIN) lens with 1 mm diameter and 4.38 mm length (GRINTECH NEM-100-25-10-860-S) was implanted at the following coordinates: AP: +1.32; ML: +1.2; DV: −4.00, 0.2 mm above the injection site. Mice were used for behavioral experiments 2–3 weeks after surgeries. For in vivo two-photon imaging experiments, mice were used no earlier than four weeks after surgery to allow sufficient and stable GCaMP6s expression. For retrograde activating DREADD experiments, 500 nl of AAV5-hSyn-Cre or AAV5-hSyn-eGFP-Cre virus were delivered bilaterally at each NAc-projecting area with following coordinates: LS (AP: 1.0, ML: ±0.4, DV: −3.5), ACA (AP: 1.0, ML: ±0.3, DV: −1.5), CLA/ECT (AP: 1.0, ML: ±2.8, DV: −3.68), mPFC (AP: 2.0, ML: ±0.4, DV: −2.5), ORB (AP: 2.6, ML: ±1.65, DV: −2.8), vSUB (AP: −4.1, ML: ±3.3, DV: −3.8), ATN (AP: −0.75, ML: ±0.9, DV: −3.15).

### Histology

Mice were transcardially perfused with PBS followed by 10% formalin or 4% PFA. Brains were dissected and post-fixed in 10% formalin or 4% PFA at 4°C overnight. Brains were sectioned into 50-μm or 100-μm coronal slices using a vibratome (Leica). For immunohistochemistry, brain sections were blocked (0.1% Triton X-100 in PBS, 3% bovine serum albumin, 2% donkey serum) and then incubated with primary antibody (rabbit anti-c-fos, Cell Signaling, 1:500 for 100-μm sections; rabbit anti-Phospho-S6, Invitrogen, 1:1000 for 50-μm sections; chicken anti-GFP, Aves labs, 1:1000 for 50-μm sections; rat anti-mCherry, Invitrogen, 1:1000 for 50-μm sections) for 2 days at 4°C. Sections were then washed and incubated with secondary antibody (donkey anti-rabbit IgG Alexa 568, Invitrogen, 1:500 for 100-μm sections; donkey anti-rabbit IgG Alexa 647, Invitrogen, 1:1000 for 50-μm sections; donkey anti-chicken IgG Alexa 488, Jackson ImmunoResearch, 1:1000 for 50-μm sections; donkey anti-rat IgG Alexa 594, Invitrogen, 1:1000 for 50-μm sections) for 1 hour at room temperature, washed again, mounted with DAPI Fluoromount-G (Southern Biotech) and imaged with the same exposure time per batch using a SlideView microscope (VS200, Olympus). Images underwent minimal processing (such as adjusting brightness and contrast) performed using ImageJ. To quantify pS6 fluorescent intensity, a circular ROI of NAc core from each section was drawn in ImageJ, and fluorescent intensity was measured by the “Measure” function from the ImageJ menu “Analyze”.

### Whole-brain FOS mapping and GFP labeling

SHIELD-based whole-brain clearing and labeling was employed for mapping FOS and GFP in mice that had received repeated exposure to 20 mg/kg cocaine or 10 mg/kg morphine vs. saline. One hour after the final injection, mice were anesthetized with isoflurane and transcardially perfused with PBS containing 10 U/ml heparin, followed by 4% PFA. The dissected brains were fixed in 4% PFA for 24 h at 4 °C. Brains were then transferred to PBS containing 0.1% sodium azide until brain clearing and labeling. Brains were processed by LifeCanvas Technologies following the SHIELD protocol as previously published (22, 83). Samples were cleared for 7 days with Clear+ delipidation buffer, followed by batch labeling in SmartBatch+ with 8.6 μg Goat anti-GFP antibody (Encor GPCA-GFP), 17 μg anti-Mouse NeuN antibody (Encor MCA-1B7), and 6 μg anti-Rabbit FOS (Abcam ab214672) per brain. Fluorescently conjugated secondary antibodies were applied in 1:2 primary/secondary molar ratios (Jackson ImmunoResearch). Labeled samples were incubated in EasyIndex (LifeCanvas Technologies) for refractive index matching (n = 1.52) and imaged with SmartSPIM (LifeCanvas Technologies) at 4 μm z-step and 1.8 μm xy pixel size. Image analysis was conducted following the procedures as previously published (22, 83). Due to technical limitations raised by resolution limitations and distinct subcellular expression patterns of FOS (nuclear) and GFP (cytosolic) signals in three-dimensional space, the direct co-localization between FOS+ and GFP+ cells were not analyzed.

Atlas registration. Samples were registered to the Allen Brain Atlas (Allen Institute: https://portal.brain-map.org/) using an automated process (alignment performed by LifeCanvas Technologies, referred to as LCT). A NeuN channel for each brain was registered to an average NeuN atlas (generated by LCT using previously registered samples). Registration was performed using successive rigid, affine, and b-spline warping algorithms (SimpleElastix: https://simpleelastix.github.io/).

Cell detection. Automated cell detection was performed by LCT using a custom convolutional neural network created with the Tensorflow python package (Google). The cell detection was performed by two networks in sequence. First, a fully convolutional detection network (84) based on a U-Net architecture (85) was used to find possible positive locations. Second, a convolutional network using a ResNet architecture (86) was used to classify each location as positive or negative. Using the previously calculated Atlas Registration, each cell location was projected onto the Allen Brain Atlas in order to count the number of cells for each atlas defined region.

For studies of acute exposure to and during spontaneous withdrawal from cocaine and morphine, brain were postfixed after perfusion as stated above, and serial 100um sections were collected between olfactory bulb and brainstem. FOS staining was conducted following standard immunohistochemistry procedures, and analysis was conducted using the open-source SMART pipeline as previously published (87).

### *In silico* FOS-Seq

FOS counts were z-scored within each batch of samples across conditions. Multiple batches of z-scored FOS activity were pooled for downstream analysis. After batch correction, images were co-registered with the Allen Brain Atlas reference mouse brain to demarcate region specificity of FOS signal. Next, normalization of FOS signal was achieved by subtracting average FOS levels in the saline condition from FOS levels in the cocaine and morphine conditions across brain regions. Region-specific gene expression data were then obtained from the Allen Brain Atlas *in situ* hybridization database, and brain areas identified to match between FOS imaging and in situ hybridization database were used for generating vectors for FOS and all identified genes. Each FOS vector and gene X ISH vector were first sigmoid transformed, followed by computations of Pearson Correlation Coefficients (PCCs) across brain regions for each condition. Our intention using the sigmoid transformation before conducting Pearson correlation was to avoid the bias resulting from outlier values that could lead to an inflation of the coefficients. These PCCs with vs. without sigmoid transformation were highly correlated in a linear manner. We recommend validating the distributions of FOS and gene X ISH vectors, as well as the “housekeeping” reference genes, in a specific biological context when applying sigmoid transformation. A histogram depicting the distribution of total PCCs was plotted. Next, a Gaussian distribution was fitted to this histogram (Fig. S5a, μ = 0.0, σ = 0.15). A threshold of ± 0.15, which corresponded to 1σ, was applied to identify genes that exhibited significant correlations. Genes correlated with PCC value > 0.15 or < −0.15 and p < 0.05 were classified as positively correlated or negatively correlated, and the rest were classified as not correlated. The method was adapted from a previously published approach (22). P-values are FDR-corrected at 5% threshold, referred to as adjusted P-values.

### snRNAseq sample preparation

Animals were stereotaxically injected with AAV-Rheb-sgRNAs or AAV-scrambled-sgRNAs bilaterally in NAc core. Notably, CRISPR-mediated DNA double-strand breaks could potentially induce general cellular effects. Thus, for future studies, it is recommended to use alternative control sgRNAs targeting a “neutral gene”, such as Rosa26. To avoid confounds from Rheb and Control sgRNAs (scrambled, non-targeting), each mouse was only transduced with one type of sgRNAs that was either Rheb-targeted or non-targeted without pooling AAVs. Three D1-Cas9 mice and three D2-Cas9 mice were transduced with Rheb-sgRNAs, and similarly three D1-Cas9 and three D2-Cas9 mice were transduced with Scrambled-sgRNAs. Three weeks after viral injections, mice were anesthetized under 5% isoflurane. NAc core was microdissected under a stereomicroscope in pre-chilled dissection buffer and immediately transferred to dry ice prior to downstream nuclei extraction. After dissection, frozen tissues on dry ice or stored in a −80°C freezer were immediately transferred to Teflon homogenizers containing 1 ml pre-chilled NP40 lysis buffer (Fisher Cat# FNN0021) and homogenized 15–30 times using a pellet pestle on ice. Homogenized samples were incubated for another 10–15 min on ice, followed by passing through a 70 μm Flowmi Cell Strainer and a 40 μm Flowmi Cell Strainer (Millipore Sigma). The collected flowthrough was centrifuged at 500–1000 rcf for 5 min at 4°C and pellets were resuspended in staining buffer. 30% iodixanol buffer was then carefully loaded at the bottom of resuspended nuclei at 4°C. Samples were centrifuged at 10000 rcf for 20 min at 4°C. Supernatant containing debris was carefully removed. Pellets were resuspended in staining buffer containing anti-NeuN Alexa 647 antibody (abcam, Cat# ab190565) in order to enrich neurons. After antibody incubation and rotating for 30 min at 4°C, samples were washed with staining buffer without antibodies for 3 times. Samples were resuspended in FACS buffer after last-round wash and sent for FACS sorting. Hoechst 33342 (ThermoFisher Scientific, Cat# H3570) were added at a final concentration of 0.2 mM to label nuclei. Sorted nuclei were sent for downstream 10X genomics 5’ RNA-seq with CRISPR library preparation and sequenced using NovaSeq sequencer with ~30000 reads/nuclei on average. Dissection buffer contains 1X HBSS, 2.5 mM HEPES-KOH [pH 7.4], 35 mM glucose, 4 mM NaHCO_3_, and actinomycin D (Sigma-Aldrich, Cat# A1410) at a final concentration of 20 μg/ml. NP40 lysis buffer contains 10 mM Tris-HCl [pH 7.4], 10 mM NaCl, 3 mM MgCl_2_, 0.1% NP40 dissolved in nuclease-free water. For 1 ml NP40 lysis buffer, 1 μl DTT, 25 μl 20 U/μl SupeRasine (Thermo Cat# AM2696), 12.5 μl 40 U/μl RNasin (Promega Cat# N2615), 10 μl protease and phosphatase inhibitor cocktail (100X; Thermo Cat# 78442), 40 μl 1 mg/ml actinomycin D were added right before use. 30% iodixanol buffer contains 0.25 M sucrose, 25 mM KCl, 5 mM MgCl_2_, 20 mM Tricine-HCl [pH 8.0] and 30% Iodixanol dissolved in nuclease-free water. DTT, Superasine, Rnasin and protease inhibitors were added at the same concentration as NP40 lysis buffer right before use. Staining buffer contains 2% BSA, 0.05% NP40 dissolved in nuclease-free 1X PBS. Superasine, Rnasin and protease inhibitors were added at the same concentration as NP40 lysis buffer right before use. FACS buffer contains 2% BSA dissolved in nuclease-free 1X PBS buffer. Superasine, Rnasin and protease inhibitors were added at the same concentration as NP40 lysis buffer immediately prior to use.

### snRNAseq analysis

Fastq files were aligned to mouse genome (mm10) and CRISPR sgRNA sequences, and expression levels in each cell were estimated with Cellranger (v 6.0.0). The gene expression count matrix for each sample was processed with the following steps: (1) Estimate doublet with Scrublet (https://github.com/swolock/scrublet) (88); (2) Estimate and correct the ambient RNA contaminations with SoupX (https://github.com/constantAmateur/SoupX) (89); (3) Load the corrected counting matrix into Seurat object with log normalization; (4) Calculate the proportion of UMIs from mitochondrial genes; and (5) The cells assigned as doublets or mitochondrial content >1% were removed. The Seurat objects were integrated by following the RPCA workflow (https://satijalab.org/seurat/articles/integration_rpca.html) (90). The number of PCs used for UMAP calculation was selected with elbow plot (91). Then, the clustering was calculated with Leiden algorithm (92). Mixscape analysis was applied following the workflow (https://satijalab.org/seurat/articles/mixscape_vignette) (26). After Mixscape classification of perturbed vs. escaped cells, only perturbed cells and control cells were used for calculating differentially expressed genes. The differential gene expression of each comparison was performed with Seurat::FindMarkers() with logfc.threshold greater than 0.13, and raw p values from the returned set of genes were corrected using p.adjust() with the ‘BH’ method. Significant differential expression genes were defined as log2 (Fold Change) greater than 0.26 or less than −0.26, and corrected p values less than 0.05. GO analyses were conducted using the gseapy.enrichr() function in Python. Databases included GO_Biological_Process_2023, GO_Cellular_Component_2023, GO_Molecular_Function_2023. Enriched pathways with adjusted p values less than 0.05 were plotted.

### Chemogenetic modulations

5 mg/kg CNO was i.p. injected 20 min prior to injections of other solutions. For experiments aimed at activating retrograde NAc-projecting neurons, 2 mg/kg CNO was i.p. injected 30 min prior to providing access to food or water. Food consumption was measured in mice that had prior ad libitum access to food, 1 hour after the onset of the dark cycle, resembling a state of physiological hunger. Water consumption was measured 5 min after providing overnight water-deprived mice with free access to water. We reported the data as % change, because animals from each of these 9 groups were tested in cohorts (i.e., at varying times) due to logistical limitations. The % change was calculated by normalizing the food intake or water intake of experimental groups to that of the control group within the same cohort test.

### Optogenetic modulations

For photostimulating ChR2, a 473-nm laser (OEM Lasers/OptoEngine) was used to generate laser pulses (5–7 mW at the output tip of the fiber, 5 ms, 20 Hz) throughout the behavioral session, except when noted otherwise, controlled by a waveform generator (Keysight).

### Feeding and drinking behaviors

For optogenetic stimulation, mice were acclimated to a new clean cage for 5 minutes before experiments started. Mice were then provided free access to either food or water. For ChR2 stimulation in fasted or water-dehydrated mice, food or water intake was measured after 20 minutes of photostimulation, followed by 20 minutes with laser off.

### Open field assay

Mice were introduced into a 28 × 28 cm open field arena. Locomotor activity and fraction of movement over the 20-minute session were automatically tracked and quantified by Ethovision 9 (Noldus).

### Real-time place preference assay

Real-time place preference was conducted in a two-chamber acrylic box (50 × 25 × 25 cm). The preference between the two chambers was determined by the time spent on either side over a 20-minute session. The amount of time spent in each chamber was automatically tracked and calculated using the Ethovision 9 software (Noldus). Photostimulation of ChR2 (5–7 mW per fiber tip; 20 Hz; 10 ms pulses; 5 s on, 5 s off) was delivered by a mini-IO box (Noldus) when mice entered the designated light-paired side. Mice performed three test sessions with photostimulation.

### *In vivo* two-photon imaging

Two-photon (2P) calcium imaging was performed on a Scientifica SliceScope galvo-scanning 2P microscope with a Nikon 16×/0.8 water-dipping objective and a Coherent Chameleon Ultra II Ti:Sapphire laser source. The objective was focused onto the rear image plane of the implanted GRIN lens, so that the excitation laser beam was relayed into the sample by the GRIN lens, and conversely fluorescence was relayed out of the sample and recorded using photon-multiplier tubes in the SliceScope’s non-descanned detection head. Microscope hardware and data acquisition was controlled using the ScanImage software (versions 5.5 and 5.6, Vidrio Technologies), which is based on Matlab (The Mathworks). The field-of-view of the microscope was sufficient to record the diameter of the GRIN lens image, 500 μm, at a frame rate of 4.82 Hz. Liquid food (Ensure) and water were provided via a spout connected to a touch detector (lickometer) during the imaging. Mice were adapted to liquid food as the only nutrition source for at least three days before the refeeding experiments. On experiment day, fasted or dehydrated mice went through a three-minute baseline recording, followed by 3 μL water or liquid food dispensed by the lickometer spout at the beginning of the consumption trial. After the trial started, the lickometer spout dispensed 3 μL water or liquid food upon each lick from mice.

### Behavioral assays with drug administration

For behavioral experiments, a dosage of 20 mg/kg cocaine, or 10 mg/kg morphine, dissolved in saline was administered via daily single-dose i.p. injections. These doses were chosen based on roughly equivalent rewarding properties in conditioned place preference studies (8). Mice were single-housed at a minimum of 3 days before the initiation of drug administration and maintained single-housed throughout the tests. Daily food and water consumption were quantified by weighing the food pellets or water bottles using a high precision scale (Bonvoisin Lab Scale) on each day and subtracting these measurements from the previous day’s (24 hours in between) measurements.

For refeeding and rehydration assays, single-housed mice were overnight fasted or water deprived for a period of 16–18 hours. On the second day before the tests, mice remained in their home cages while new food pellets or water bottles were provided 20 min after i.p. injections of cocaine or morphine. Food or water consumption was assessed by weighing the food pellets or water bottles before and after consumption over a cumulative period of 30 min, 2 hours, and 4 hours. Behavioral tests during the spontaneous withdrawal were conducted within 2 days after 24 hours post last drug exposure.

During the 2-day spontaneous withdrawal period, refeeding and rehydration assays were each conducted once on each day and randomized for different cohorts of mice. During the spontaneous withdrawal, each group of mice received daily i.p. injections of saline.

The sucrose preference test was conducted once during the spontaneous withdrawal period. These three groups of naïve animals underwent habituation with the sucrose preference test before drug exposure, followed by another sucrose preference test after 5-day repeated drug exposure or saline treatment. These mice were water deprived overnight before the test. During the test, mice received two bottles, one containing 2% sucrose solution, the other containing water. The sucrose preference test comprised two rounds, each round allowed each animal 5 min of free access to both solutions. To mitigate bias towards a particular side, the water and sucrose bottles were swapped between sides during the second round.

For *in vivo* two-photon calcium imaging experiments, a dosage of 10 mg/kg cocaine or 5 mg/kg morphine dissolved in saline was administered by single-dose i.p. injections. Notably, doses in the calcium imaging experiments were lowered to minimize excessive treadmill running which produce movement-related artefacts, while maintaining high enough doses that are known to still be rewarding (8, 93). On the experimental day, mice were head-fixed and placed on the treadmill for 5 min habituation. Mice went through a one-minute baseline recording, followed by one i.p. injection of saline, cocaine or morphine. The neural responses of each mouse were then recorded for 1 minute every 10 minutes within one hour. All cohorts of mice received one daily i.p. injection of cocaine or morphine continuing for 5 days. Mice were able to freely walk on the treadmill, and velocity was recorded at the same time. Mice had *ad libitum* access to food and water before and after the imaging, but were restricted throughout experimental procedures.

### Data Analysis

FOS-mapping data were analyzed by z-score transformation of FOS counts from each batch of the data. To generate the clustered heatmap, we then conducted one-way ANOVA on the z-scored FOS levels for each brain region across the three groups and identified brain regions with statistically significant (p < 0.05) changes for downstream K-means clustering analysis. Notably, we did not normalize the FOS counts into the density, because each brain region volume was the same for each sample as the FOS counts were projected onto the Allen brain atlas, and our statistical tests were conducted within each brain region, not across different brain regions. To compare the similarity of FOS levels across phases of drug exposure, we calculated the Euclidean distance between two vectors for each brain region, which consists of z-scored FOS expression values corresponding to either cocaine conditions (acute, withdrawal, chronic) or morphine conditions (acute, withdrawal, chronic). Both of these vectors were normalized against z-scored FOS expression values from the saline condition (acute, withdrawal, chronic) by subtraction.

Behavior data were analyzed using the Ethovision X9 software (Noldus). For two-photon imaging experiments, behavioral and imaging data were analyzed using the Suite2p pipeline (94) and custom Python scripts. Multiple trials within the same session were first concatenated, then processed by the Suite2p pipeline with non-rigid motion correction, to extract all neurons recorded across trials. According to the Suite2p pipeline, this concatenation does not normalize or alter the raw fluorescence of individual trials. Each session data from different days were processed independently. Neurons extracted by the pipeline were subsequently curated manually in the Suite2p graphical user interface. When aiming to compare responses of the same neurons across multiple sessions or days, spatial footprints of neurons from each session extracted by suite2p were mapped using CellReg (95). Consistent with prior reports (96), 20–50% of the same neurons were able to be tracked across multiple daily sessions.

To preprocess the data from food and water gel sensory responses, total fluorescence of each individual neuron was normalized using the formula z = (Fraw – μ)/σ, where Fraw is the raw fluorescence as extracted by the Suite2p pipeline, μ is the mean of Fraw during the baseline period (30 seconds prior to food and water gel presentation), and σ is the standard deviation of Fraw during the baseline period.

The data from liquid food and water consumption experiments were preprocessed as follows in order to reduce variabilities for multiple-day comparisons: The raw fluorescence was z-scored according to the formula z = (F– μ)/σ, where F is calculated from the raw fluorescence by applying a second order Butterworth filter with normalized cut-on frequency 0.266; μ is the mean baseline fluorescence (taken over the traces with activity below the median during the baseline period), σ is the standard deviation of fluorescence during the baseline period (taken over the traces with activity below the median during the baseline period). For this analysis, neurons with averaged responses larger than 3σ from 10 seconds after consumption start were considered to be activated.

Clustered neural traces during consumption were identified by k-means clustering. The neuronal states based on the k-means clustering label were projected to two-dimensional space using the t-SNE algorithm or non-negative matrix factorization (NMF) for visualization.

To preprocess the data from the drugs of abuse experiments, the raw fluorescence traces were filtered as described above. Within each session, each 1-minute imaging trace was normalized as Fcorr = (F– μ)/μ, where F is the raw fluorescence and μ is the average of the lowest quintile of the raw fluorescence. All corrected traces were concatenated and used for downstream analyses. Peaks of neuronal responses were identified using the SciPy package (find_peak() function) with the peak height threshold set to 3σ) and minimum distance between peaks set to ~1.3 seconds (based on the GCaMP6s decay time). Strength of neuronal preference was defined as: (Peak_cocaine_ – Peak_food/water_)/(Peak_cocaine_ + Peak_food/water_). To quantify cocaine-activated neurons (peak greater than 3σ), the timespan from 20–40 minutes post i.p. injection was used, while 30–50 minutes post i.p. injection was used to quantify morphine-activated neurons. The timespan was chosen based on observed behavioral effects (Fig. S1), neuronal dynamics (Fig. S4), and established pharmacokinetics from literature for cocaine/morphine post i.p. injections (97, 98).

To differentiate the motor-associated from the non-motor-associated neural correlates, we first converted the animals’ walking velocity into binary movement vectors (moving vs. not moving) as recorded from the treadmill and then computed the cross-correlation (evaluated for a range of 1–10 frames lag, corresponding to ~2 seconds) between neuronal traces and binary movement vectors (as computed by thresholding the raw velocity measurement vector). The lag with the highest cross-correlation was chosen for downstream analyses. We next computed the Pearson correlation coefficients (PCC) between the lag-corrected neuronal traces and the binary movement vector. We selected highly motion-correlated neuronal traces as those with the modulus of their PCC with the movement vector above a threshold in the range 0.2–0.4. We adjusted the threshold based on the principle that the averaged frames of neural activities (above median) during movement periods are no less than those during the non-movement period, Wilcoxon test was conducted to compare the significant difference level between the movement versus non-movement frames. The neurons showing PCCs larger than the threshold were considered to be motor-associated neurons, the rest were nonmotor-associated neurons. Each neuronal pair with activity correlation coefficient greater than 0.3 was considered to be “synchronized”. A connectivity index was then calculated as the total number of pairs of synchronized neurons divided by the pairs of synchronized neurons during the baseline period (before i.p. injection).

Tensor component analysis (TCA) was conducted as described previously (21). The tensor matrix comprised of trial, neuron and time series of neuronal activity was loaded into the TCA algorithm. The factor matrices were constrained to be nonnegative. Each neural state was averaged from multiple sessions recorded from each cohort of mice. Neurons positively contributing to state 1 relative to state 2 were determined by positive values after the subtraction: state 1 loadings – state 2 loadings.

### Statistics and reproducibility

Statistical analyses were conducted using Graphpad Prism 10.1. Throughout the paper, values are reported as mean ± s.e.m. (error bar or shaded area). P-values for pair-wise comparisons were obtained using the two-tailed Wilcoxon signed-rank test or two-tailed paired Student’s t-test. P-values for comparisons across independent groups or multiple groups were conducted using two-tailed independent Student’s t-test, or ANOVA (with repeated measures when possible) and corrected for multiple comparisons. The statistical models used for imaging data analysis as described above were carried out using the scikit-learn Python package (99). No statistical methods were used to predetermine sample size. The experiments were not randomized. The investigators were not blinded to allocation during experiments and outcome assessment. Representative images were selected from 3 to 5 original biological replicates.

## Supplementary Material

Supplementary Material

MDAR checklist

## Data Availability

All data are available in the manuscript and supplementary material. Additional datasets and custom analysis codes necessary to understand the conclusions are deposited at Dryad (100).
