# Supplementary material for: Drugs of abuse hijack a mesolimbic pathway that processes homeostatic need
Source: Science. Author manuscript; Available in PMC 2025 Apr 19. (PMC11077477; doi:10.1126/science.adk6742)
Supplement: Supplementary Material [file NIHMS1987613-supplement-Supplementary_Material.pdf]

Science
AAAS

# Supplementary Materials for

## Drugs of abuse hijack a mesolimbic pathway that processes homeostatic need

Bowen Tan[1†], Caleb J. Browne[2,3†], Tobias Nöbauer[4†], Alipasha Vaziri[4,5*], Jeffrey M. Friedman[1*] & Eric J. Nestler[2*]

[†]These authors contributed equally to this work.
* Correspondence to: eric.nestler@mssm.edu; friedj@rockefeller.edu; vaziri@rockefeller.edu

**This PDF file includes:**

Figs. S1 to S10

**Other Supplementary Materials for this manuscript include the following:**

MDAR Reproducibility Checklist

# Figure S1

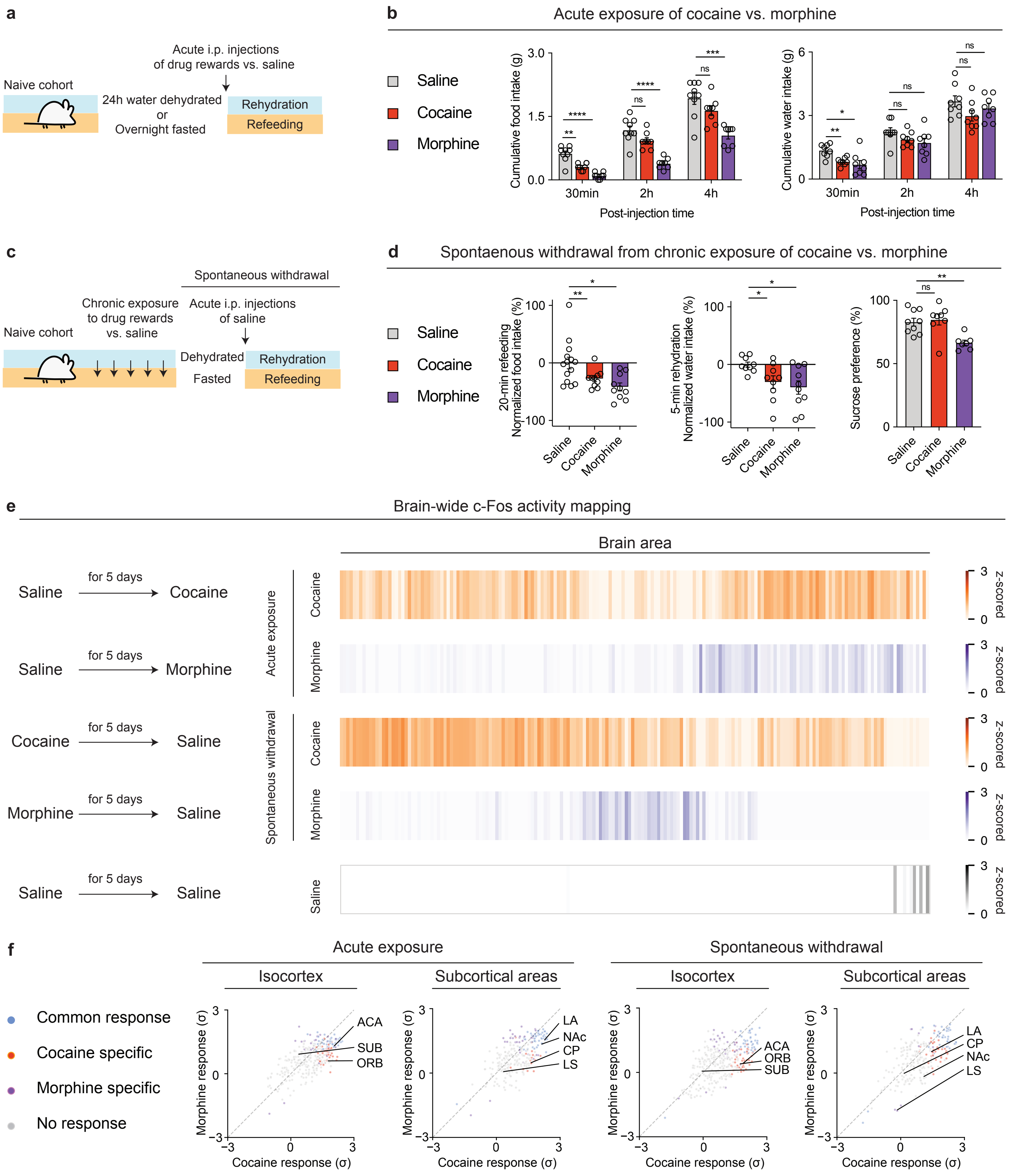

a
Acute i.p. injections
of drug rewards vs. saline
Naive cohort
24h water dehydrated
or
Overnight fasted
Rehydration
Refeeding
b
Acute exposure of cocaine vs. morphine
Saline
Cocaine
Morphine
Cumulative food intake (g)
Cumulative water intake (g)
30min
2h
4h
Post-injection time
c
Spontaneous withdrawal
Chronic exposure
to drug rewards
vs. saline
Acute i.p. injections
of saline
Naive cohort
Dehydrated
Fasted
Rehydration
Refeeding
d
Spontaneous withdrawal from chronic exposure of cocaine vs. morphine
Saline
Cocaine
Morphine
20-min refeeding
Normalized food intake (%)
5-min rehydration
Normalized water intake (%)
Sucrose preference (%)
e
Brain-wide c-Fos activity mapping
Brain area
Saline
for 5 days
Cocaine
Saline
for 5 days
Morphine
Cocaine
for 5 days
Saline
Morphine
for 5 days
Saline
Saline
for 5 days
Saline
Acute exposure
Spontaneous withdrawal
z-scored
f
Acute exposure
Spontaneous withdrawal
Isocortex
Subcortical areas
Isocortex
Subcortical areas
Common response
Cocaine specific
Morphine specific
No response
Morphine response (σ)
Cocaine response (σ)
ACA
SUB
ORB
LA
NAc
CP
LS
ACA
ORB
SUB
LA
CP
NAc
LS

**Fig. S1. Acute exposure to, and spontaneous withdrawal from, cocaine vs. morphine both reduce natural reward consumption while activating common and distinct brain areas.** (a), Schematic of the experimental design for refeeding and water rehydration assays post acute exposure to 20 mg/kg cocaine, 10 mg/kg morphine vs. saline. Comparisons of (b), Cumulative food intake in fasted mice (left panel), Cumulative water intake in water-dehydrated mice (right panel) received i.p. injections of saline, cocaine, morphine 20 min prior to providing free access to food or water (n = 9, 8, 8 for each group, respectively, two-way ANOVA with Dunnett's multiple comparisons). (c), Schematic of the experimental design of refeeding and water rehydration assays following spontaneous withdrawal from repeated exposure to 20 mg/kg cocaine, 10 mg/kg morphine vs. saline. Comparisons of (d), Normalized food intake in refeeding assay from fasted mice following spontaneous withdrawal from saline, cocaine, morphine (n = 14, 10, 10 for each group, one-way ANOVA with Dunnett's T3 multiple comparisons, data were pooled from 2 cohorts, left panel). Normalized water intake in rehydration assay from water-dehydrated mice following spontaneous withdrawal from cocaine, morphine vs. saline (n = 9, 9, 10 for each group, data were pooled from 3 cohorts, one-way ANOVA with Dunnett's T3 multiple comparisons, data were pooled from 2 cohorts, middle panel). Comparisons of sucrose preference vs. water (%) in water-dehydrated mice following spontaneous withdrawal (n = 9, 8, 7 for saline, cocaine, morphine group, respectively, one-way ANOVA with Dunnett's T3 multiple comparisons, right panel). (e), Heatmap overview of brain areas showing significantly differential FOS levels in response to acute 20 mg/kg cocaine, acute 10 mg/kg morphine, spontaneous withdrawal from repeated exposure to cocaine and morphine vs. saline (n = 3, 4, 4, 4, 5 for each group, one-way ANOVA for each brain area with cut-off $p < 0.05$ classified as statistically significant, followed by K-means clustering). (f), Scatter plot of FOS levels after acute exposure to cocaine vs. morphine (left). Scatter plot of FOS levels of following spontaneous withdrawal from cocaine vs. morphine (right). All error bars represent mean ± s.e.m. NS, not significant, *P < 0.05, **P < 0.01, ***P < 0.001, ****P < 0.0001.

# Figure S2

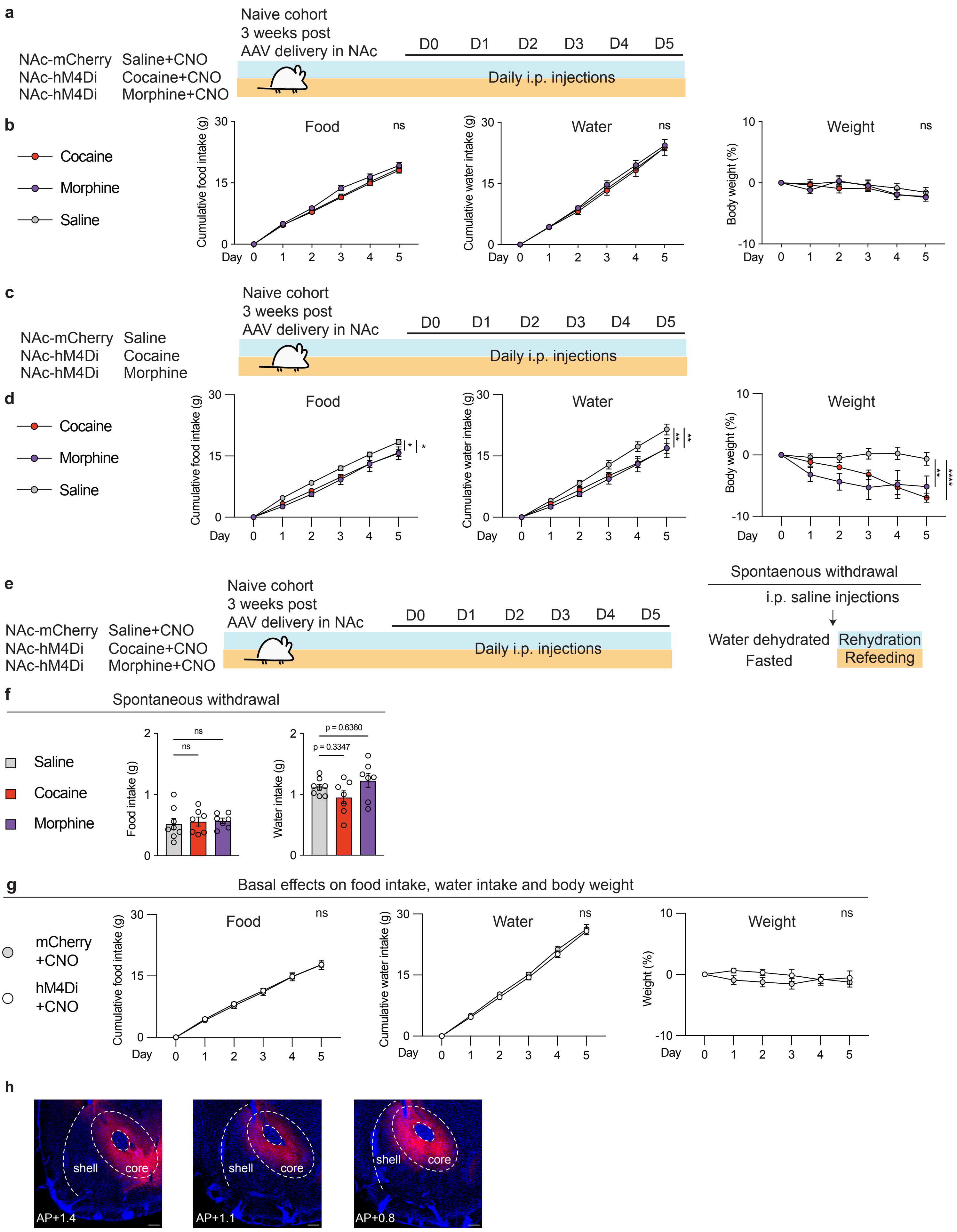

a
Naive cohort
3 weeks post
AAV delivery in NAc
D0 D1 D2 D3 D4 D5
NAc-mCherry Saline+CNO
NAc-hM4Di Cocaine+CNO
NAc-hM4Di Morphine+CNO
Daily i.p. injections
b
Cocaine
Morphine
Saline
Food
Water
Weight
ns
Cumulative food intake (g)
Cumulative water intake (g)
Body weight (%)
Day
c
Naive cohort
3 weeks post
AAV delivery in NAc
D0 D1 D2 D3 D4 D5
NAc-mCherry Saline
NAc-hM4Di Cocaine
NAc-hM4Di Morphine
Daily i.p. injections
d
Cocaine
Morphine
Saline
Food
Water
Weight
Cumulative food intake (g)
Cumulative water intake (g)
Body weight (%)
Day
e
Naive cohort
3 weeks post
AAV delivery in NAc
D0 D1 D2 D3 D4 D5
NAc-mCherry Saline+CNO
NAc-hM4Di Cocaine+CNO
NAc-hM4Di Morphine+CNO
Daily i.p. injections
Spontaneous withdrawal
i.p. saline injections
Water dehydrated
Fasted
Rehydration
Refeeding
f
Spontaneous withdrawal
Saline
Cocaine
Morphine
Food intake (g)
Water intake (g)
ns
p = 0.6360
p = 0.3347
g
Basal effects on food intake, water intake and body weight
mCherry +CNO
hM4Di +CNO
Food
Water
Weight
ns
Cumulative food intake (g)
Cumulative water intake (g)
Weight (%)
Day
h
shell
core
AP+1.4
AP+1.1
AP+0.8

**Fig. S2. Chemogenetic silencing of NAc neurons prevents attenuated natural reward consumption induced by repeated exposure to cocaine or morphine.** (a), Schematic of the experimental design for chemogenetic modulations of NAc neurons. Three groups of mice respectively expressing mCherry, hM4Di or hM4Di in NAc received 5 mg/kg CNO i.p injections 20 min prior to receiving drug rewards vs. saline for 5 days. (b), Comparisons of Cumulative food intake (g), Cumulative water intake (g), Weight (%) in NAc-mCherry + saline + CNO, NAc-hM4Di + 20 mg/kg cocaine + CNO, NAc-hM4Di + 10 mg/kg morphine + CNO, respectively (n = 8, 7, 7 for each group, two-way ANOVA with Dunnett's multiple comparisons). (c), Schematic of the experimental design for the control of chemogenetic modulations of NAc neurons. Three groups of mice respectively expressing mCherry, hM4Di or hM4Di in NAc received saline, cocaine, morphine, respectively. (d), Comparisons of Cumulative food intake (g), Cumulative water intake (g), Weight (%) in NAc-mCherry + saline, NAc-hM4Di + 20 mg/kg cocaine, NAc-hM4Di + 10 mg/kg morphine, respectively (n = 5, 7, 5 for each group, two-way ANOVA with Dunnett's multiple comparisons). (e), Schematic of the experimental design for the chemogenetic silencing. Three groups of mice expressing mCherry, hM4Di or hM4Di, respectively, received daily i.p. injections of 5 mg/kg CNO 20 min prior to receiving i.p. injections of saline, 20 mg/kg cocaine, 10 mg/kg morphine for 5 days. (f), Comparisons of Food intake (left panel), Water intake (right panel) in fasted mice or water-dehydrated mice during spontaneous withdrawal from saline, cocaine, morphine (n = 8, 7, 7 for each group, one-way ANOVA with Dunnett's T3 multiple comparisons). (g), Comparisons of Cumulative food intake, Cumulative water intake, Weight of mice expressing mCherry vs. hM4Di received daily i.p. injections of 5 mg/kg CNO for 5 days (n = 8, 7 for each group, two-way ANOVA with Šídák's multiple comparisons). (h), Example images of AAV-hM4D(Gi)-mCherry expression in the NAc core (scale bar: 200 μm) from anterior to posterior (AP) with the following coordinates: AP +1.4, +1.1, +0.8. All error bars represent mean ± s.e.m. NS, not significant, *P < 0.05, **P < 0.01, ***P < 0.001, ****P < 0.0001.

# Figure S3

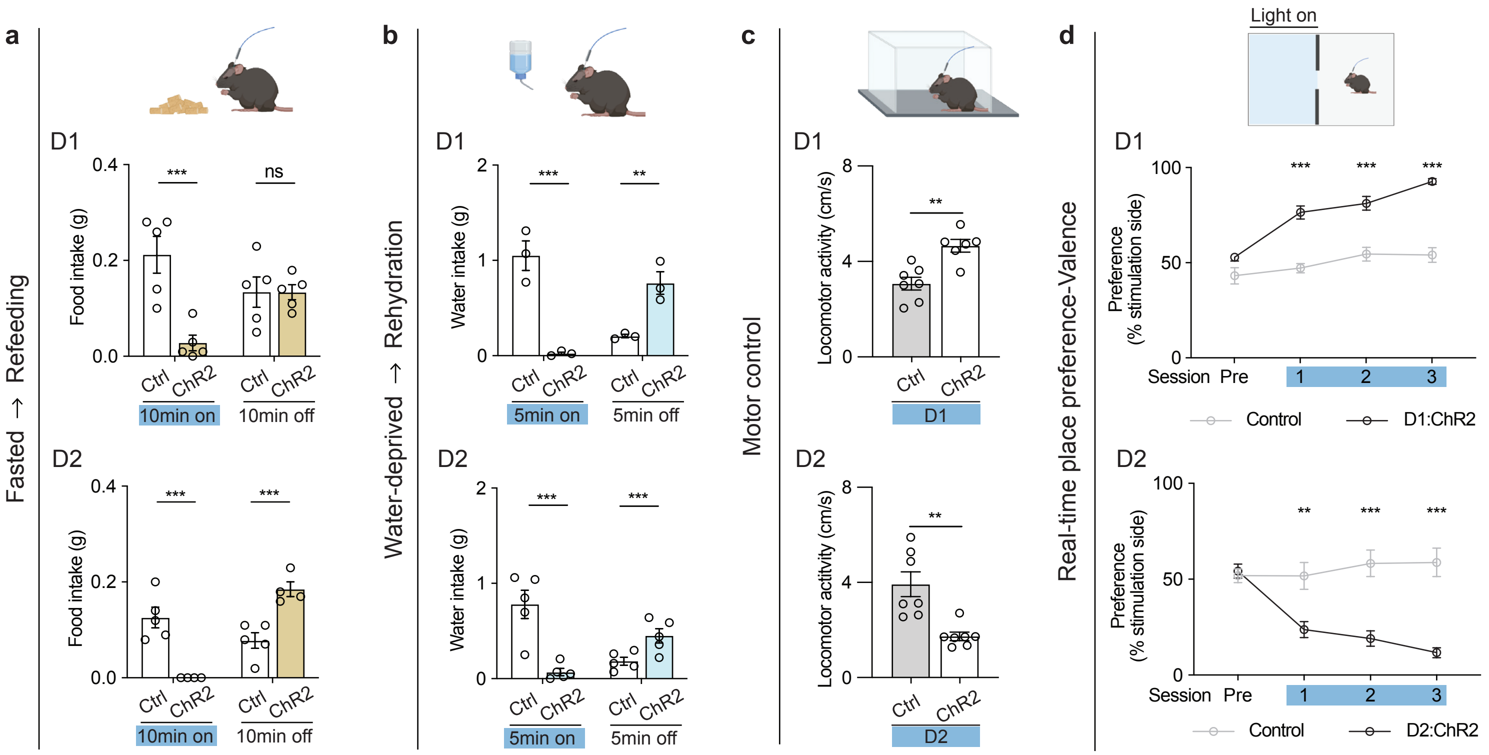
a
Fasted → Refeeding
D1
Food intake (g)
***
ns
Ctrl
ChR2
10min on
10min off
D2
***
***
b
Water-deprived → Rehydration
D1
Water intake (g)
***
**
5min on
5min off
D2
***
***
c
Motor control
D1
Locomotor activity (cm/s)
**
D2
**
d
Light on
Real-time place preference-Valence
D1
Preference (% stimulation side)
Session
Pre
Control
D1:ChR2
D2
D2:ChR2

**Fig. S3. Optogenetic activation of NAc neurons regulates key behavioral domains of natural reward processing.** (a), Schematic of the refeeding assay in fasted mice that underwent optogenetic activations of D1 or D2 MSNs in the NAc core vs. the control groups. Comparisons of food intake in Control vs. D1-ChR2 group (n = 5, 5 for each group, two-way ANOVA with Šídák's multiple comparisons, top panel). Comparisons of food intake in Control vs. D2-ChR2 group (n = 5, 4 for each group, two-way ANOVA with Šídák's multiple comparisons, bottom panel). (b), Schematic of the rehydration assay in water-dehydrated mice that underwent optogenetic activations of D1 or D2 MSNs in the NAc core vs. the control groups. Comparisons of water intake in Control vs. D1-ChR2 group (n = 3, 3 for each group, two-way ANOVA with Šídák's multiple comparisons, top panel). Comparisons of water intake in Control vs. D2-ChR2 group (n = 5, 5 for each group, two-way ANOVA with Šídák's multiple comparisons, bottom panel). (c), Schematic of the open field test in ad libitum fed mice that underwent optogenetic activations of D1 or D2 MSNs in the NAc core vs. the control groups. Comparisons of locomotor activity in the Control vs. D1-ChR2 group (n = 7, 6 for each group, two-tailed Student's t tests, top panel). Comparisons of locomotor activity in the Control vs. D2-ChR2 group (n = 7, 7 for each group, two-tailed Student's t tests, bottom panel). (d), Schematic of real-time place preference assay in ad libitum fed mice that underwent optogenetic activations of D1 or D2 MSNs in the NAc core vs. the control groups. Comparisons of the preference at the stimulation side in the Control vs. D1-ChR2 group (n = 5, 5 for each group, two-way ANOVA with Šídák's multiple comparisons, top panel). Comparisons of the preference at the stimulation side in the Control vs. D2-ChR2 group (n = 5, 6 for each group, two-way ANOVA with Šídák's multiple comparisons, bottom panel). Conditions where mice received 473 nm laser on are highlighted in blue. All error bars represent mean ± s.e.m. NS, not significant, $^*P < 0.05$, $^{**}P < 0.01$, $^{***}P < 0.001$, $^{****}P < 0.0001$.

# Figure S4

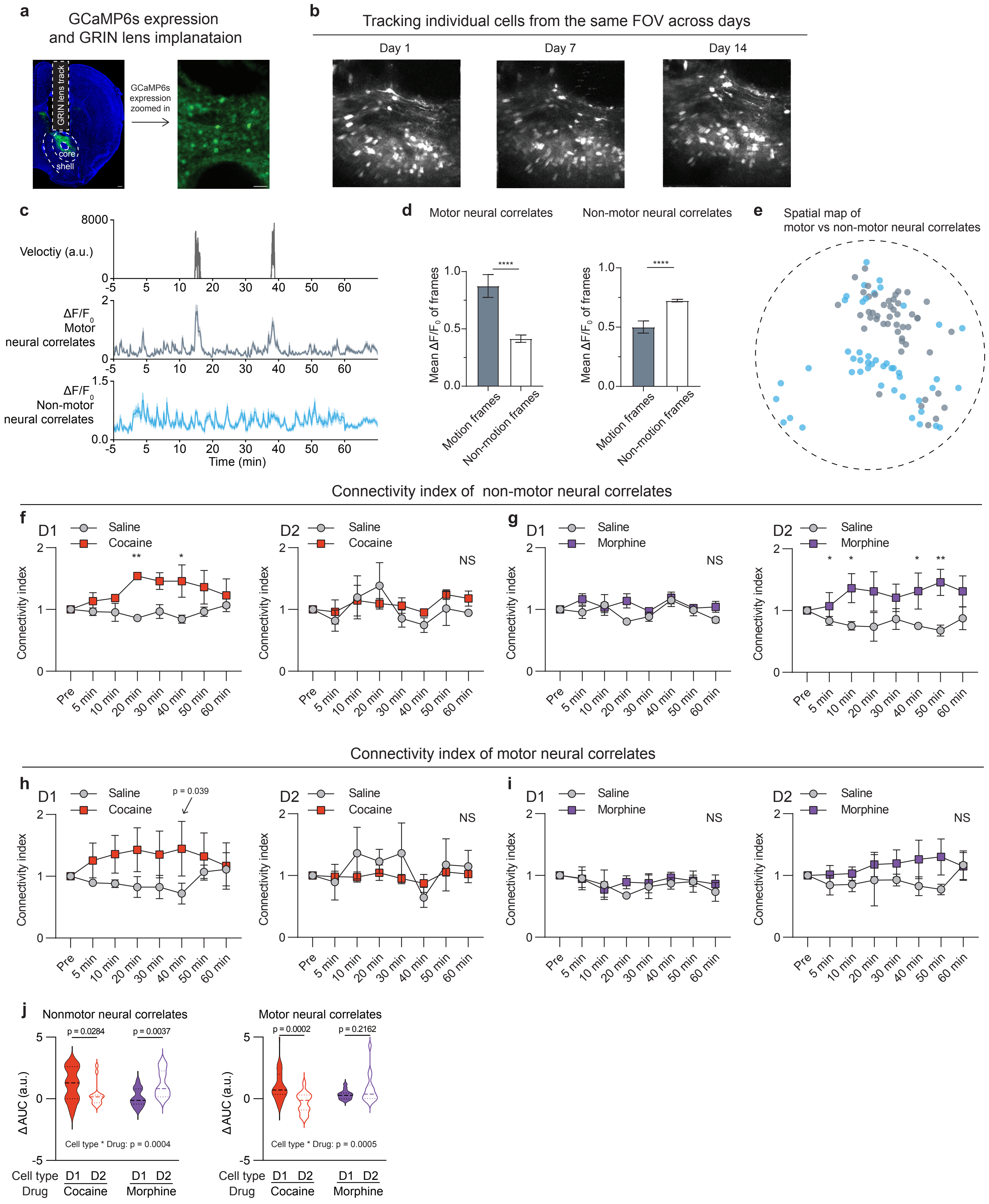

a
GCaMP6s expression and GRIN lens implanataion
GRIN lens track
core
shell
GCaMP6s expression zoomed in
b
Tracking individual cells from the same FOV across days
Day 1
Day 7
Day 14
c
Velocity (a.u.)
ΔF/F0 Motor neural correlates
ΔF/F0 Non-motor neural correlates
Time (min)
d
Motor neural correlates
Non-motor neural correlates
Mean ΔF/F0 of frames
Motion frames
Non-motion frames
e
Spatial map of motor vs non-motor neural correlates
Connectivity index of non-motor neural correlates
f
D1
D2
Saline
Cocaine
NS
g
Morphine
Connectivity index
Pre
5 min
10 min
20 min
30 min
40 min
50 min
60 min
Connectivity index of motor neural correlates
h
p = 0.039
i
j
Nonmotor neural correlates
Motor neural correlates
p = 0.0284
p = 0.0037
p = 0.0002
p = 0.2162
ΔAUC (a.u.)
Cell type * Drug: p = 0.0004
Cell type * Drug: p = 0.0005
Cell type
Drug
Cocaine
Morphine

**Fig. S4. Cocaine vs. morphine selectively synchronizes D1 or D2 MSNs neuronal firing patterns.** (a), Example image of GCaMP6s expression (scale bar: 200 μm) and GRIN lens track in the NAc core after viral transduction (left). A magnification of the GCaMP6s expression (scale bar: 50 μm) under the GRIN lens track (right). (b), Example field of views (FOV) across days. (c), Example velocity traces vs. averaged neural traces of motor- vs. nonmotor-associated neurons. (d), Example comparison of movement frames vs. nonmovement frames of motor- (n = 39 neurons) vs. nonmotor-associated (n = 92 neurons) neurons (two-tailed Wilcoxon test). (e), Example spatial distribution of motor- vs. nonmotor-associated neurons in the imaging focal plane. (d), Quantification of connectivity index averaged from all sessions of D1 nonmotor neural correlates modulated by cocaine compared to saline (n = 3 mice, two-way ANOVA, with Sidak's multiple comparisons). (left panel), Quantification of connectivity index averaged from all sessions of D2 nonmotor neural correlates modulated by cocaine compared to saline (n = 3 mice, two-way ANOVA, with Sidak's multiple comparisons) (right panel). (f), Quantification of connectivity index averaged from all sessions of D1 nonmotor neural correlates modulated by morphine compared to saline (n = 3 mice, two-way ANOVA, with Sidak's multiple comparisons, left panel), Quantification of connectivity index averaged from all sessions of D2 nonmotor neural correlates modulated by morphine compared to saline (n = 3 mice, two-way ANOVA, with Sidak's multiple comparisons, right panel). (g), Quantification of connectivity index averaged from all sessions of D1 motor neural correlates modulated by cocaine compared to saline (n = 3 mice, two-way ANOVA, with Sidak's multiple comparisons, left panel). Quantification of connectivity index averaged from all sessions of D2 motor neural correlates modulated by cocaine compared to saline (n = 3 mice, two-way ANOVA, with Sidak's multiple comparisons, right panel). (h), Quantification of connectivity index averaged from all sessions of D1 motor neural correlates modulated by morphine compared to saline (n = 3 mice, two-way ANOVA, with Sidak's multiple comparisons, left panel). (i), Quantification of connectivity index averaged from all sessions of D2 motor neural correlates modulated by morphine compared to saline (n = 3 mice, two-way ANOVA, with Sidak's multiple comparisons, right panel). (j), Δ Area under the curve (AUC) of D1 or D2 nonmotor (left) or motor (right) neural correlates in response to cocaine or morphine (n = 15 sessions pooled from 3 mice with 5 sessions per mouse. Two-way ANOVA, with Fisher's LSD tests.). All error bars represent mean ± s.e.m. NS, not significant, *P < 0.05, **P < 0.01, ***P < 0.001, ****P < 0.0001.

# Figure S5

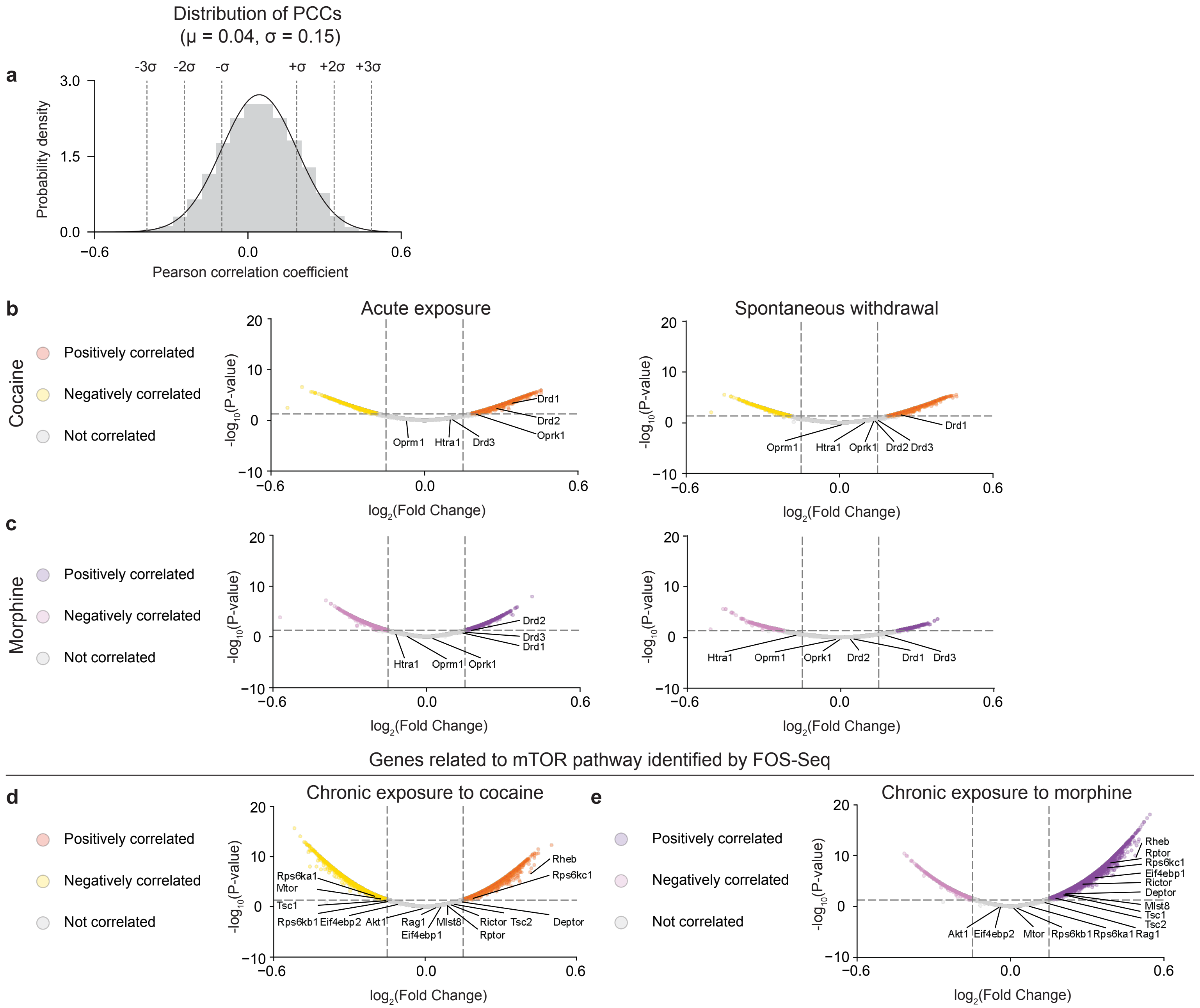

a
Distribution of PCCs
(μ = 0.04, σ = 0.15)
-3σ -2σ -σ +σ +2σ +3σ
Probability density
Pearson correlation coefficient
b
Acute exposure
Spontaneous withdrawal
Cocaine
Positively correlated
Negatively correlated
Not correlated
Drd1 Drd2 Oprk1 Oprm1 Htra1 Drd3
c
Morphine
Positively correlated
Negatively correlated
Not correlated
Genes related to mTOR pathway identified by FOS-Seq
d
Chronic exposure to cocaine
e
Chronic exposure to morphine
Rheb Rps6kc1 Rps6ka1 Mtor Tsc1 Rps6kb1 Eif4ebp2 Akt1 Rag1 Mlst8 Eif4ebp1 Rictor Tsc2 Rptor Deptor
-log10(P-value)
log2(Fold Change)

**Fig. S5.** ***In silico*** **FOS-Seq identifies canonical marker genes associated with acute exposure to, or spontaneous withdrawal from, cocaine vs. morphine.** (a), Histogram distribution of Pearson correlation coefficients (PCC) pooled from all the data after FOS-Seq. Volcano plot of genes correlated with (b), acute exposure to cocaine (left panel), spontaneous withdrawal from cocaine (right panel). (c), acute exposure to morphine (left panel), spontaneous withdrawal from morphine (right panel). (d), Volcano plot of genes in the mTOR pathway correlated with repeated exposure to cocaine. (e), Volcano plot of genes in the mTOR pathway correlated with repeated exposure to morphine. Genes with Pearson Correlation Coefficient > 0.15 or < -0.15, and $p < 0.05$ are classified as Positively correlated or Negatively correlated, the rest are classified as Not correlated.

# Figure S6

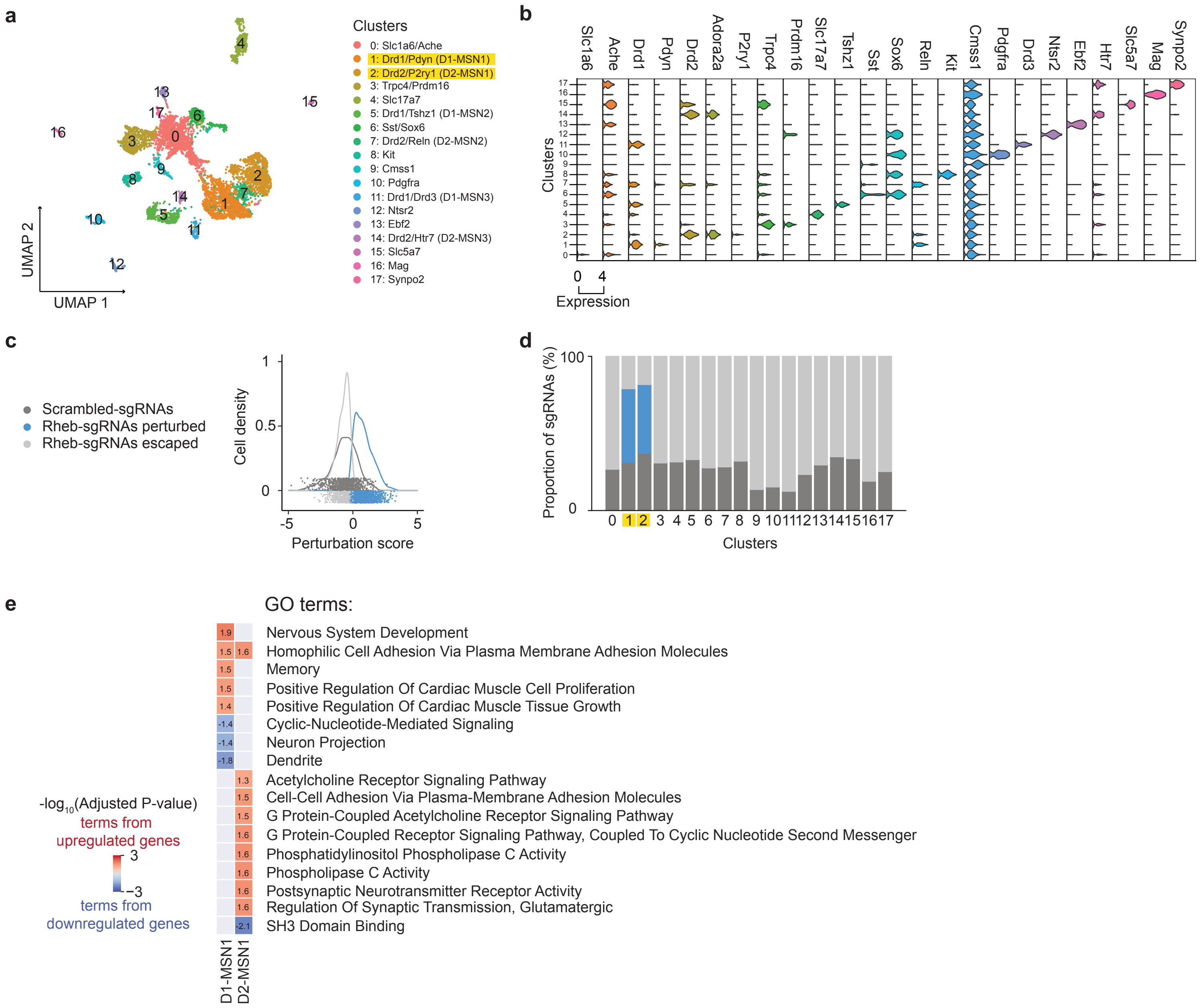
a
Clusters
0: Slc1a6/Ache
1: Drd1/Pdyn (D1-MSN1)
2: Drd2/P2ry1 (D2-MSN1)
3: Trpc4/Prdm16
4: Slc17a7
5: Drd1/Tshz1 (D1-MSN2)
6: Sst/Sox6
7: Drd2/Reln (D2-MSN2)
8: Kit
9: Cmss1
10: Pdgfra
11: Drd1/Drd3 (D1-MSN3)
12: Ntsr2
13: Ebf2
14: Drd2/Htr7 (D2-MSN3)
15: Slc5a7
16: Mag
17: Synpo2
UMAP 1
UMAP 2
b
Slc1a6 Ache Drd1 Pdyn Drd2 Adora2a P2ry1 Trpc4 Prdm16 Slc17a7 Tshz1 Sst Sox6 Reln Kit Cmss1 Pdgfra Drd3 Ntsr2 Ebf2 Htr7 Slc5a7 Mag Synpo2
Clusters
0 4
Expression
c
Scrambled-sgRNAs
Rheb-sgRNAs perturbed
Rheb-sgRNAs escaped
Cell density
Perturbation score
d
Proportion of sgRNAs (%)
Clusters
e
GO terms:
Nervous System Development
Homophilic Cell Adhesion Via Plasma Membrane Adhesion Molecules
Memory
Positive Regulation Of Cardiac Muscle Cell Proliferation
Positive Regulation Of Cardiac Muscle Tissue Growth
Cyclic-Nucleotide-Mediated Signaling
Neuron Projection
Dendrite
Acetylcholine Receptor Signaling Pathway
Cell-Cell Adhesion Via Plasma-Membrane Adhesion Molecules
G Protein-Coupled Acetylcholine Receptor Signaling Pathway
G Protein-Coupled Receptor Signaling Pathway, Coupled To Cyclic Nucleotide Second Messenger
Phosphatidylinositol Phospholipase C Activity
Phospholipase C Activity
Postsynaptic Neurotransmitter Receptor Activity
Regulation Of Synaptic Transmission, Glutamatergic
SH3 Domain Binding
-log10(Adjusted P-value)
terms from upregulated genes
terms from downregulated genes
D1-MSN1
D2-MSN1

**Fig. S6. snRNAseq after CRISPR knockout of *Rheb* reveals cell-type-specific transcriptional regulation.** (a), UMAP distribution of 18 clusters. (b), Stacked violin plot of marker gene expressions for each cluster. (c), Distribution of individual cell's perturbation scores. (d), Composition of Rheb-sgRNAs perturbed, Rheb-sgRNAs escaped and the control Scrambled-sgRNAs across each cluster. (e), Significant GO terms from the differentially expressed genes of D1-MSN1 vs. D2-MSN1 (Adjusted P-value < 0.05).

## Figure S7

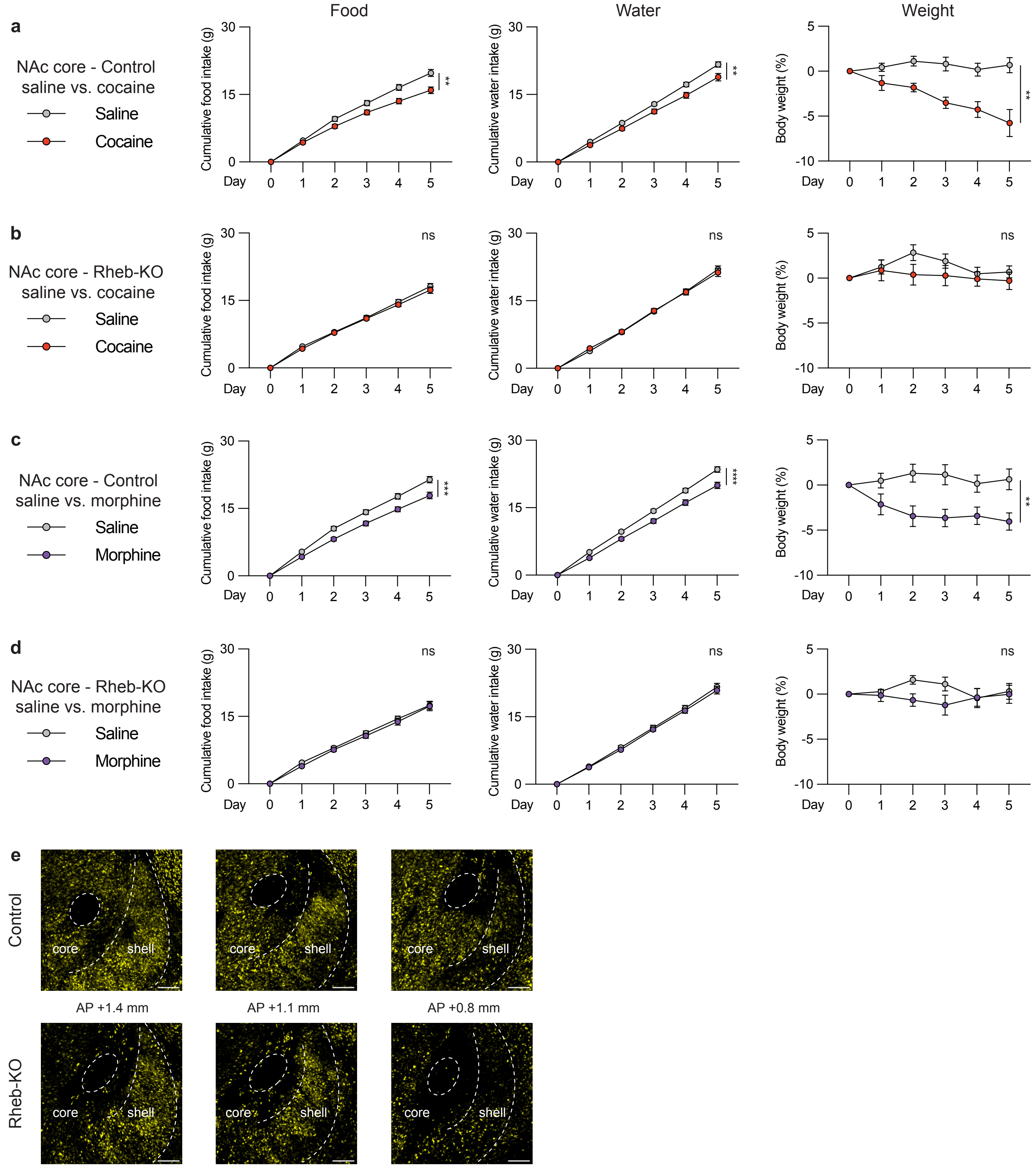

a
b
c
d
e
Food
Water
Weight
NAc core - Control
saline vs. cocaine
NAc core - Rheb-KO
saline vs. cocaine
NAc core - Control
saline vs. morphine
NAc core - Rheb-KO
saline vs. morphine
Saline
Cocaine
Morphine
Cumulative food intake (g)
Cumulative water intake (g)
Body weight (%)
Day
ns
Control
Rheb-KO
core
shell
AP +1.4 mm
AP +1.1 mm
AP +0.8 mm

**Fig. S7. *Rheb* knockout in the NAc prevents the suppression of natural reward consumption induced by repeated exposure to cocaine or morphine.** (a), Comparisons of time-course cumulative food intake (g), water intake (g), weight (%) in the Control group treated with saline for 5 days followed by another 5-day cocaine treatment (n =10, 10 for each group). (b), Comparisons of time-course cumulative food intake (g), water intake (g), weight (%) in the Rheb knockout (Rheb-KO) group treated with saline for 5 days followed by another 5-day cocaine treatment (n = 8, 8 for each group). (c), Comparisons of time-course cumulative food intake (g), water intake (g), weight (%) in the Control group treated with saline for 5 days followed by another 5-day morphine treatment (n = 10, 10 for each group). (d), Comparisons of time-course cumulative food intake (g), water intake (g), weight (%) in the Rheb knockout (Rheb-KO) group treated with saline for 5 days followed by another 5-day morphine treatment (n = 8, 8 for each group). Two-way ANOVA with Šídák's multiple comparisons were used to compare saline vs. each drug treatment. The 5-day daily averaged food or water intake, and weight after 5-day treatment are presented in Fig. 5n, o. (e), Example images of pS6 expression in the NAc core (scale bar: 200 μm) from anterior to posterior with the following coordinates: AP +1.4, +1.1, +0.8 for both the Control and Rheb-KO groups. All error bars represent mean ± s.e.m. NS, not significant, $^{*}P < 0.05$, $^{**}P < 0.01$, $^{***}P < 0.001$, $^{****}P < 0.0001$.

# Figure S8

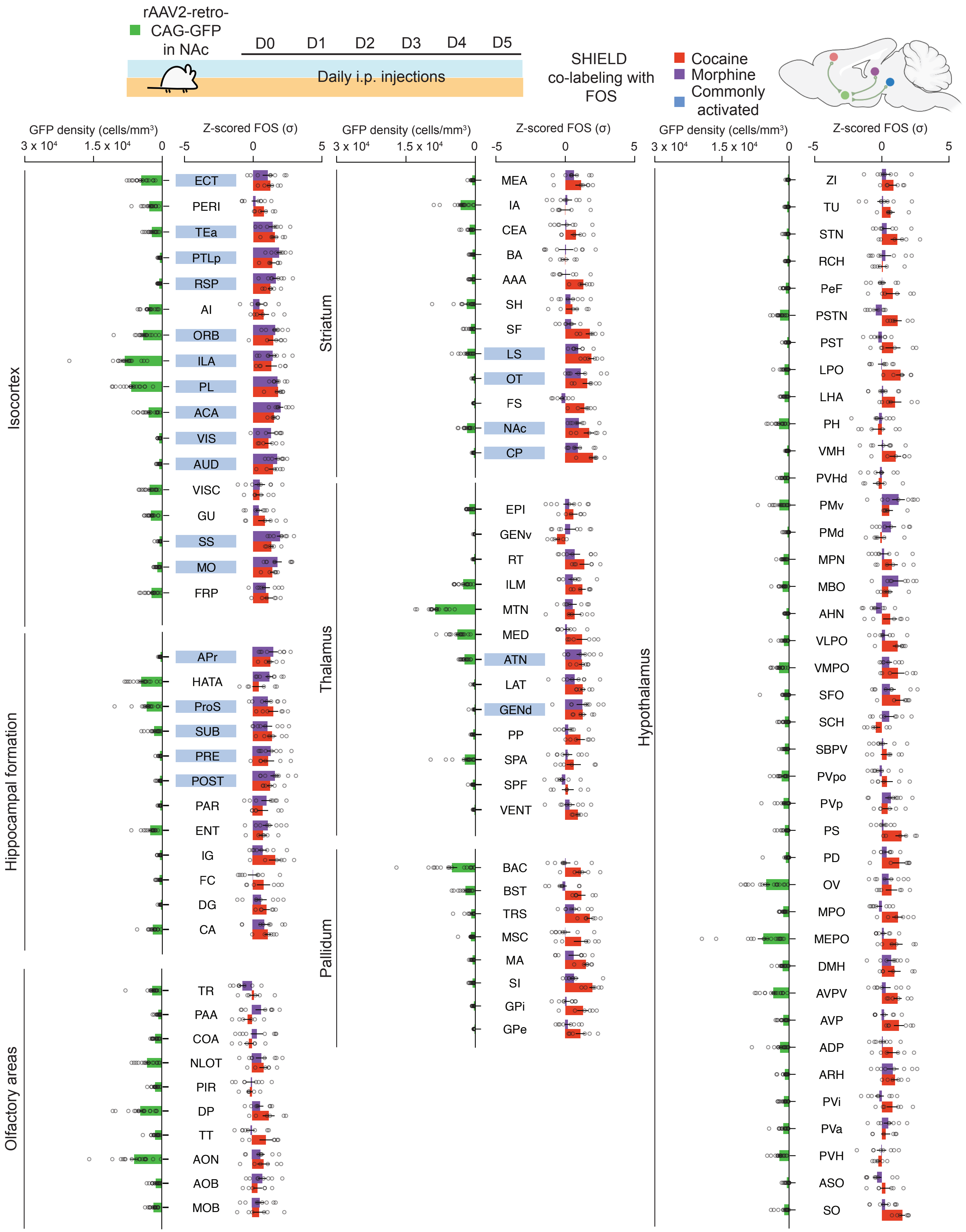

rAAV2-retro-CAG-GFP in NAc
D0
D1
D2
D3
D4
D5
Daily i.p. injections
SHIELD co-labeling with FOS
Cocaine
Morphine
Commonly activated
GFP density (cells/mm³)
Z-scored FOS (σ)
3 x 10⁴
1.5 x 10⁴
0
-5
0
5
Isocortex
ECT
PERI
TEa
PTLp
RSP
AI
ORB
ILA
PL
ACA
VIS
AUD
VISC
GU
SS
MO
FRP
Hippocampal formation
APr
HATA
ProS
SUB
PRE
POST
PAR
ENT
IG
FC
DG
CA
Olfactory areas
TR
PAA
COA
NLOT
PIR
DP
TT
AON
AOB
MOB
Striatum
MEA
IA
CEA
BA
AAA
SH
SF
LS
OT
FS
NAc
CP
Thalamus
EPI
GENv
RT
ILM
MTN
MED
ATN
LAT
GENd
PP
SPA
SPF
VENT
Pallidum
BAC
BST
TRS
MSC
MA
SI
GPi
GPe
Hypothalamus
ZI
TU
STN
RCH
PeF
PSTN
PST
LPO
LHA
PH
VMH
PVHd
PMv
PMd
MPN
MBO
AHN
VLPO
VMPO
SFO
SCH
SBPV
PVpo
PVp
PS
PD
OV
MPO
MEPO
DMH
AVPV
AVP
ADP
ARH
PVi
PVa
PVH
ASO
SO

**Fig. S8. Simultaneous FOS mapping and monosynaptic retrograde tracing from NAc in response to cocaine vs. morphine.** Schematic of experimental design for retrograde tracing from NAc and repeated exposure to drug rewards. Three cohorts of mice with rAAV2-retro-CAG-GFP delivered in NAc received daily i.p. injections of 20 mg/kg cocaine, 10 mg/kg morphine and saline for 5 days (Top panel). GFP density and FOS activity in each brain area (n = 17 for GFP density pooled from n = 6 mice received saline, n = 6 mice received cocaine, n = 5 mice received morphine, FOS activity is pooled from multiple batches and normalized by z-score with n = 6 mice received cocaine, n = 8 mice received morphine followed by subtraction of averaged z-score from n = 9 saline control, bottom panel). All error bars represent mean ± s.e.m. NS, not significant, *P < 0.05, **P < 0.01, ***P < 0.001, ****P < 0.0001.

# Figure S9

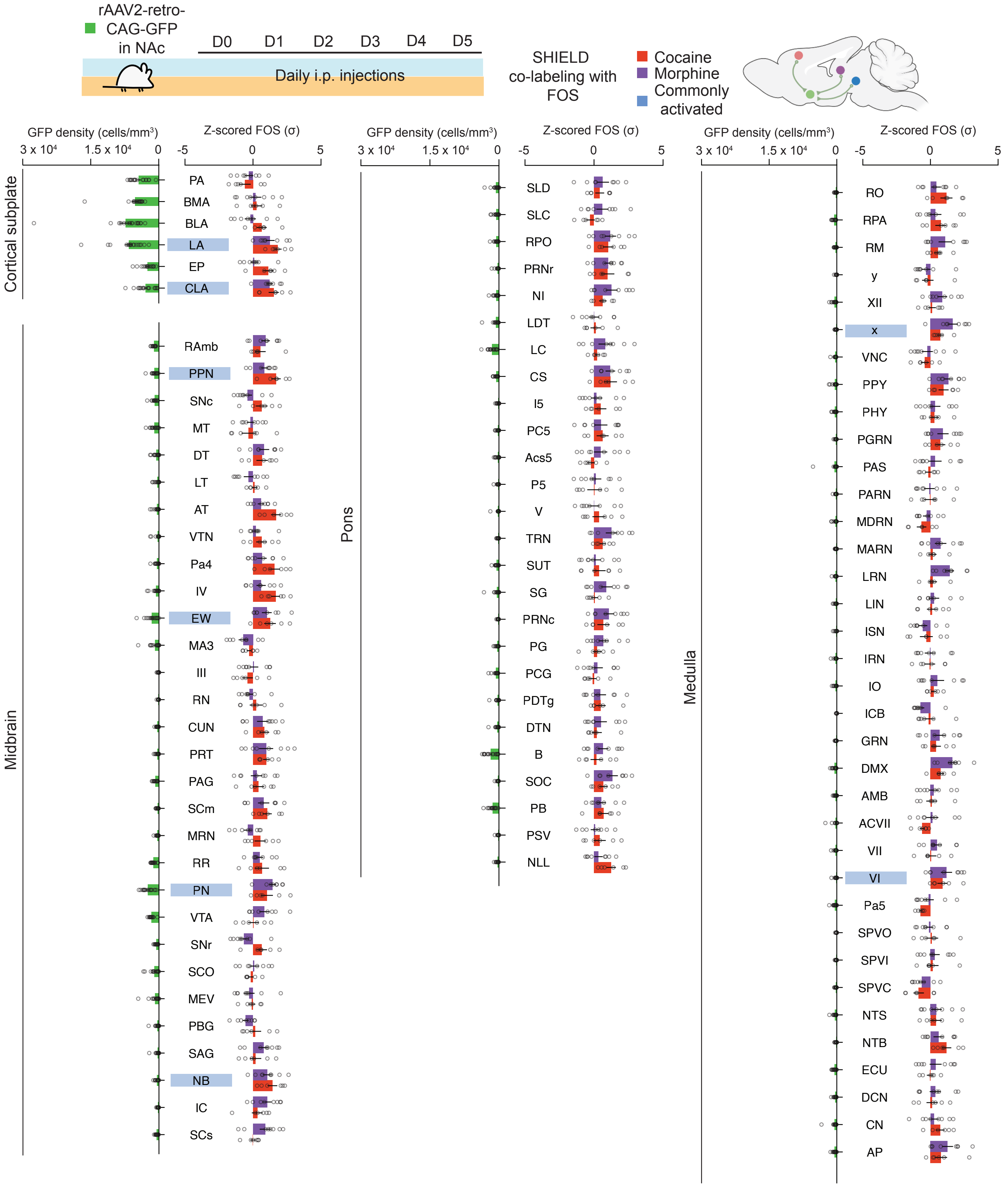

rAAV2-retro-CAG-GFP in NAc
D0
D1
D2
D3
D4
D5
Daily i.p. injections
SHIELD co-labeling with FOS
Cocaine
Morphine
Commonly activated
GFP density (cells/mm³)
Z-scored FOS (σ)
3 x 10⁴
1.5 x 10⁴
0
-5
0
5
Cortical subplate
PA
BMA
BLA
LA
EP
CLA
Midbrain
RAmb
PPN
SNc
MT
DT
LT
AT
VTN
Pa4
IV
EW
MA3
III
RN
CUN
PRT
PAG
SCm
MRN
RR
PN
VTA
SNr
SCO
MEV
PBG
SAG
NB
IC
SCs
Pons
SLD
SLC
RPO
PRNr
NI
LDT
LC
CS
I5
PC5
Acs5
P5
V
TRN
SUT
SG
PRNc
PG
PCG
PDTg
DTN
B
SOC
PB
PSV
NLL
Medulla
RO
RPA
RM
y
XII
x
VNC
PPY
PHY
PGRN
PAS
PARN
MDRN
MARN
LRN
LIN
ISN
IRN
IO
ICB
GRN
DMX
AMB
ACVII
VII
VI
Pa5
SPVO
SPVI
SPVC
NTS
NTB
ECU
DCN
CN
AP

**Fig. S9. (Continued from Figure S8) Simultaneous FOS mapping and monosynaptic retrograde tracing from NAc in response to cocaine vs. morphine.** Schematic of experimental design for retrograde tracing from NAc and repeated exposure to drug rewards. Three cohorts of mice with rAAV2-retro-CAG-GFP delivered in NAc received daily i.p. injections of 20 mg/kg cocaine, 10 mg/kg morphine and saline for 5 days (top panel). GFP density and FOS activity in each brain area ($n = 17$ for GFP density pooled from $n = 6$ mice received saline, $n = 6$ mice received cocaine, $n = 5$ mice received morphine, FOS activity is pooled from multiple batches and normalized by z-score with $n = 6$ mice received cocaine, $n = 8$ mice received morphine followed by subtraction of averaged z-score from $n = 9$ saline control, bottom panel). All error bars represent mean ± s.e.m. NS, not significant, *$P < 0.05$, **$P < 0.01$, ***$P < 0.001$, ****$P < 0.0001$.

# Figure S10

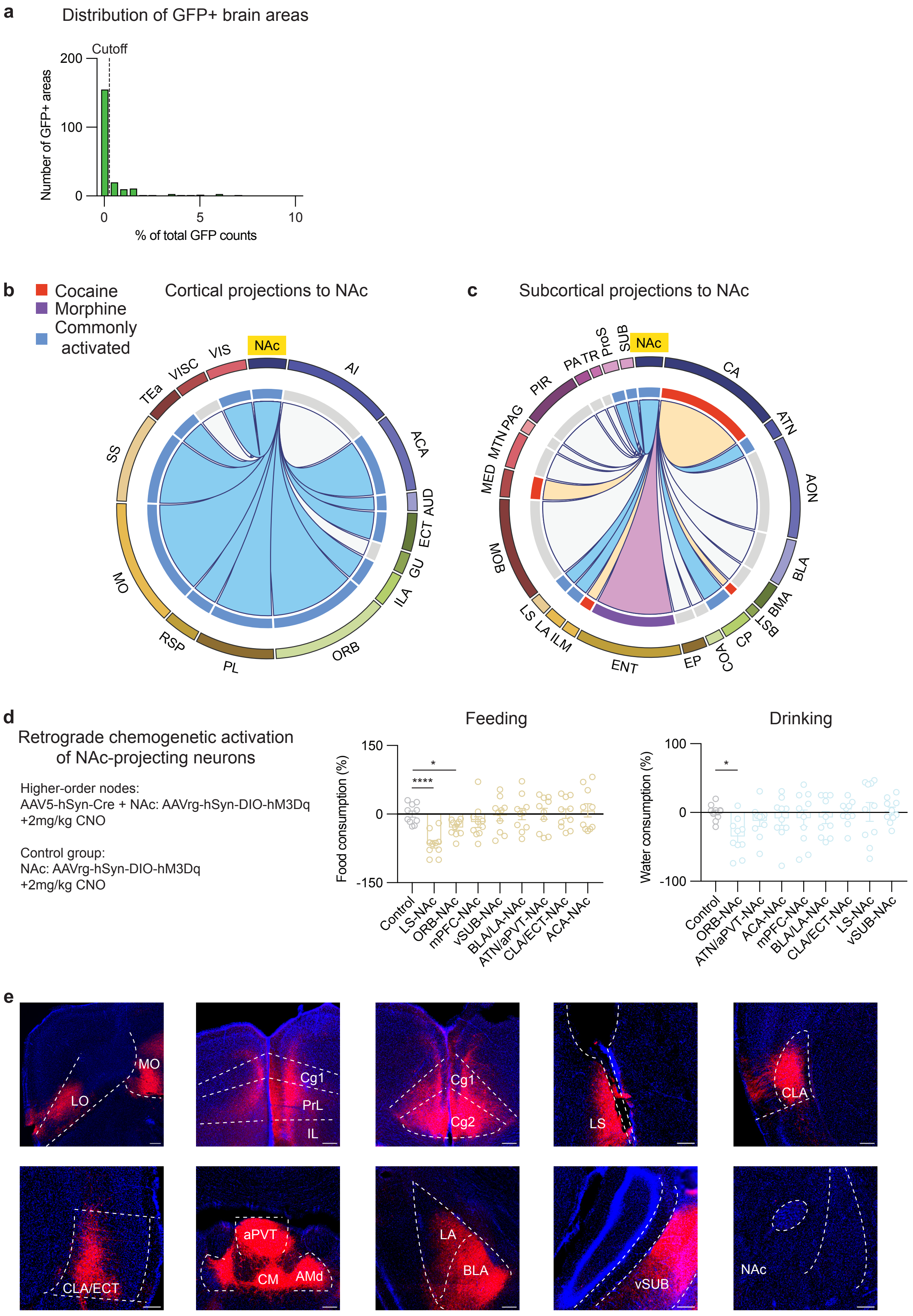
a
Distribution of GFP+ brain areas
Cutoff
Number of GFP+ areas
% of total GFP counts
b
Cocaine
Morphine
Commonly activated
Cortical projections to NAc
NAc
AI
ACA
AUD
ECT
GU
ILA
ORB
PL
RSP
MO
SS
TEa
VISC
VIS
c
Subcortical projections to NAc
SUB
ProS
TR
PA
PIR
PAG
MTN
MED
MOB
LS
LA
ILM
ENT
EP
COA
CP
BST
BMA
BLA
AON
ATN
CA
d
Retrograde chemogenetic activation of NAc-projecting neurons
Higher-order nodes:
AAV5-hSyn-Cre + NAc: AAVrg-hSyn-DIO-hM3Dq
+2mg/kg CNO
Control group:
NAc: AAVrg-hSyn-DIO-hM3Dq
+2mg/kg CNO
Feeding
Food consumption (%)
Control
LS-NAc
ORB-NAc
mPFC-NAc
vSUB-NAc
BLA/LA-NAc
ATN/aPVT-NAc
CLA/ECT-NAc
ACA-NAc
Drinking
Water consumption (%)
Control
ORB-NAc
ATN/aPVT-NAc
ACA-NAc
mPFC-NAc
BLA/LA-NAc
CLA/ECT-NAc
LS-NAc
vSUB-NAc
e
MO
LO
Cg1
PrL
IL
Cg1
Cg2
LS
CLA
CLA/ECT
aPVT
CM
AMd
LA
BLA
vSUB
NAc

**Fig. S10. Brain-wide distribution of cocaine- and morphine-activated brain areas that project to NAc.** (a), Histogram distribution of the number of GFP+ brain areas vs. percentage of total GFP counts. GFP+ brain areas with %GFP counts > 0.5% are used to generate the following circos maps (b), A circos map summarizing cortical projections to the NAc. (c), A circos map summarizing subcortical projections to the NAc. (d), Retrograde chemogenetic activation of NAc-projecting neurons in each ascending node identified from the above circos maps. Food or water consumption was measured 30 min after i.p. injections of 2 mg/kg CNO for both the Control and other hM3Dq-expressing groups. (n = 10 for each group of mice. One-way ANOVA, with Dunnett's T3 multiple comparisons). (e), Example images of AAVrg-DIO-hM3Dq-mCherry expression in each of the NAc-projecting brain region and the NAc of the Control group (scale bar: 200 μm). All error bars represent mean ± s.e.m. NS, not significant, *P < 0.05, **P < 0.01, ***P < 0.001, ****P < 0.0001.
